# Analysis of Multiple *Brachyspira hyodysenteriae* Genomes Confirms That the Species Is Relatively Conserved but Has Potentially Important Strain Variation

**DOI:** 10.1371/journal.pone.0131050

**Published:** 2015-06-22

**Authors:** Michael Black, Paula Moolhuijzen, Roberto Barrero, Tom La, Nyree Phillips, David Hampson, Werner Herbst, Stefanie Barth, Matthew Bellgard

**Affiliations:** 1 Centre for Comparative Genomics, Murdoch University, Murdoch, Western Australia, Australia; 2 School of Veterinary and Life Sciences, Murdoch University, Murdoch, Western Australia, Australia; 3 Institute for Hygiene and Infectious Diseases of Animals, Justus-Liebig University Giessen, Giessen, Germany; University of Minnesota, UNITED STATES

## Abstract

The intestinal spirochete *Brachyspira hyodysenteriae* is an important pathogen in swine, causing mucohemorrhagic colitis in a disease known as swine dysentery. Based on the detection of significant linkage disequilibrium in multilocus sequence data, the species is considered to be clonal. An analysis of the genome sequence of Western Australian *B*. *hyodysenteriae *strain WA1 has been published, and in the current study 19 further strains from countries around the world were sequenced with Illumina technology. The genomes were assembled and aligned to over 97.5% of the reference WA1 genome at a percentage sequence identity better than 80%. Strain regions not aligned to the reference ranged between 0.2 and 2.5%. Clustering of the strain genes found on average 2,354 (88%) core genes, 255 (8.6%) ancillary genes and 77 (2.9%) unique genes per strain. Depending on the strain the proportion of genes with 100% sequence identity to WA1 ranged from 85% to 20%. The result is a global comparative genomic analysis of *B*. *hyodysenteriae *genomes revealing potential differential phenotypic markers for numerous strains. Despite the differences found, the genomes were less varied than those of the related pathogenic species *Brachyspira pilosicoli*, and the analysis supports the clonal nature of the species. From this study, a public genome resource has been created that will serve as a repository for further genetic and phenotypic studies of these important porcine bacteria. This is the first intra-species *B*. *hyodysenteriae* comparative genomic analysis.

## Introduction

The intestinal spirochete *Brachyspira hyodysenteriae* is the etiological agent of swine dysentery (SD)—a severe mucohemorrhagic colitis of pigs [1] Despite the economic importance of SD and the need to control the disease, knowledge is lacking about metabolic and other adaptations that have allowed the spirochete to successfully colonize the complex and potentially hostile environment of the large intestine, and to induce disease [2]. Similarly, precise virulence mechanisms remain poorly understood. Key genes of interest are those that have been linked with virulence, including those associated with chemotaxis, motility, accessory factors for substrate utilization, lipoproteins and hemolysins [3–5].

Currently there are sequence data available for 341 *Brachyspira hyodysenteriae* strains in the PubMLST multilocus sequence typing (MLST) database (http://pubmlst.org/brachyspira/). Analysis of these MLST data has revealed a significant linkage disequilibrium in *B*. *hyodysenteriae*, implying that it is essentially a clonal species [6–8], whilst in contrast the related pathogenic intestinal spirochete *Brachyspira pilosicoli* forms a diverse recombinant species [9]. Moreover, in the first *Brachyspira* species multi-strain genome comparison, three strains of *B*. *pilosicoli* were shown to vary considerably in their genome size and organisation, with extensive recombination being present [10]. To date no similar multi-strain analysis of *B*. *hyodysenteriae* genomes has been conducted.

In this study we produced a pan-genome based analysis and report comparative genomics for the 20 *B*. *hyodysenteriae* strains in order to identify any clades and hyper-variable and conserved genomic regions. In evaluating these genomes we aimed to identify key differences in the 19 newly sequenced strains in relation to the completed reference genome strain WA1 [2], and to compare the extent of genomic variation in *B*. *hyodysenteriae* strains compared to the variation previously found in *B*. *pilosicoli*.

## Material and Methods

### Genomic Sequence Source

An analysis of the genome sequence of *B*. *hyodysenteriae* genome reference strain WA1 (3,000,694 base pairs) used in this study was published in 2009 (GenBank accessions NC_012225 and NC_012226) [2]. This strain contained a single circular chromosome and a ~36 Kb plasmid. In the current study new genomic sequences from re-sequenced WA1 and another 19 *B*. *hyodysenteriae* strains from different geographic areas (Table 1) were obtained from Boehringer Ingelheim, who had undertaken sequencing of these genomes using Illumina technology as part of a collaboration with the authors. The original strains that were sequenced came from the collections at Reference Centre for Intestinal Spirochetes at Murdoch University and from the collection at Justus Liebig University Giessen. All strains came from pigs with swine dysentery. Strains B78^T^, B6933, FM88.90 and G21 have been shown to be less virulent than some other strains in experimentally infected pigs [11,12]. The sequence data were obtained as FASTA read sequences, except for strain FM88.90, which were received in the form of assembled contigs. The genome sequences have been deposited in GenBank in the following bioproject: http://www.ncbi.nlm.nih.gov/bioproject/PRJNA272555/. Accession numbers for each isolate are shown in Table 1. Genomic data for the four publically available *B*. *pilosicoli* strains (95/1000; WesB; B2904; P43/6/78^T^) were downloaded from NCBI.

**Table 1:** *Brachyspira hyodysenteriae* strain names, origin and GenBank accession numbers for the genomes.

Strain name	Country of isolation (Australian State)	GenBank accession number
865	Korea	JXNA00000000
FMV89.3323	Canada	JXNB00000000
NX	Not recorded	JXNC00000000
B204	USA	JXND00000000
B6933	USA	JXNE00000000
B78^T^	USA	JXNF00000000
B8044	USA	JXNG00000000
G21	Germany	JXNH00000000
G44	Germany	JXNI00000000
FM88.90	Canada	JXNJ00000000
NSW15	Australia (New South Wales)	JXNK00000000
NSW5	Australia (New South Wales)	JXNL00000000
Q17	Australia (Queensland)	JXNM00000000
ST190	Germany	JXNN00000000
ST195	Germany	JXNO00000000
ST210	Japan	JXNP00000000
ST265	Spain	JXNQ00000000
Vic2	Australia (Victoria)	JXNR00000000
WA100	Australia (Western Australia)	JXNS00000000

### Genome assembly and annotation

Eighteen of the 19 *B*. *hyodysenteriae* strain genomes were assembled in the bioinformatics resource Yabi [13] using Velvet [14] with an optimised kmer size of 53. Strain FM88.90 was assembled with SeqMan NGen Assembly 3.1.2 build 5, and set parameters of genome length 3,200,000 bp, max gap of 6, match size: 21, match spacing of 50 and minimum match percent of 93. Vector contamination was screened with true vectScan [15].

All genomes were annotated using RAST [16] with frame shift error correction selected for gene predictions and gene annotation. The WA1 reference genome (chromosome and plasmid) was re-annotated using the same pipeline in order to eliminate any variables from using differing annotation pipelines.

### Genome alignment to the reference WA1 genome

Genome and plasmid contiguous sequences were aligned to the reference NC_012225 and NC_012226 sequence accessions respectively using MUMMER version 3, NucMER [17], with coords and ‘mumreference’ options, then sequence average identities calculated on aligned sequence.

### Gene alignment to WA1 reference genome and plasmid

RAST nucleotide gene predictions for the strains were aligned to the reference NC_012225 and NC_012226 sequence accessions using BLAT [18] at 90% identity and 90% coverage. The alignments were then visualised in the Integrative Genomics Viewer (IGV) [19].

### Gene alignment relative to WA1 reference genes

Strain RAST nucleotide gene predictions were compared to the reference genes using BLAT at greater than 50 percentage sequence identity and greater than 90% coverage to estimate the distribution of full-length genes by percentage identity. The full-length genes (90% coverage) were grouped at 50, 60, 70, 80, 90, 95 and 100% sequence identity.

### Gene protein clustering

Gene protein predictions were clustered using BLAST MCL version 08–312 at an expected Blastp value of 1e-20 and an inflation rate of 2.5 to identify protein clusters.

### Protein Blast Matrix

A BLASTMatrix [20] protein analysis for the19 strains plus the reference was completed at 50% coverage and an expected value of 1e-06, based on generating pair-wise reciprocal Blastp [21] identity percentages for homologous genes and a “self” Blastp to identify paralogous proteins within each strain. The matrix was not expected to be symmetrical as differing identities for the same pair-wise homologous match may occur in reciprocal Blastp depending on which proteome is the target and which is the query.

### Phylogenetics

MLST was conducted as previously described [22]. The genes analysed encoded alcohol dehydrogenase (*adh*), alkaline phosphatase (*alp*), esterase (*est*), glutamate dehydrogenase (*gdh*), glucose kinase (*glpK*), phosphoglucomutase (*pgm*), and acetyl-CoA acetyltransferase (*thi*). They were concatenated in order for each strain and then each of these concatenated sequences was compiled into a multi-FASTA file. This was then subject to ClustalW [23] alignment. A maximum likelihood tree was calculated from this alignment with MEGA5 [24] The maximum likelihood method was chosen as it has previously been shown to be the preferred phylogenetic methodology for intra-species comparisons [25]. Using the Akaike Information Criterion (AIC), the best model was selected, which was the general time reversible (GTR) model with gamma distribution and invariant sites. This was then utilised to calculate a bootstrap consensus maximum likelihood tree compiled from 100 bootstrap iterations.

### Analysis of selected gene families

Selected gene families of interest were extracted from each strain via BLAT alignment with the reference WA1 GenBank database. EMBOSS tools [26] were used to extract and collate FASTA files of gene families for each strain as well as construct multi-strain MUSCLE [27] alignments of each gene. Neighbour-joining trees for each gene were constructed via Newick Tools [28].

## Results and Discussion

### Strain genome assembly

All strains had pair end short read FASTA sequences of 78 base lengths available, except for FM88.90 which had 100 base pair end Illumina Hi-Seq reads. The sequence data set construct and estimated sequencing depth of coverage for the 20 strains are shown in Table 2. The short read assemblies had a kmer depth of coverage based on assembly that ranged from 88 to 272. The final number of assembled contigs for all the strains ranged from 124 to 279. The genome assembly statistics in Table 2 show that the genome size for all strains was ~3 Mb (2,995–3,175kb), which is in agreement with the size of the reference *B*. *hyodysenteriae* WA1 genome [2]. The variation in genome size was less than for *B*. *pilosicoli*, where the four sequenced strains WesB, B2904, P43/6/78^T^ and 95/1000 had genome sizes of 2,890, 2,765, 2,556 and 2,596 Kb, respectively [10, 29, 30].

**Table 2:** *Brachyspira hyodysenteriae* strain sequence information, assembly statistics and gene content.

	Isolate	n	n:N50	Min	Median	Mean	N50	Max	Sum	Coverage	Proteins	Fl-gene	tRNA	rRNA
1	865	174	9	105	369	17,351	111,229	320,126	3,019,141	214	2,594	2,165	33	3
2	FMV89.33232	170	6	105	350	18,675	142,799	367,712	3,174,794	171	2,795	2,210	33	3
3	NX	212	11	105	390	14,252	78,384	196,663	3,021,518	188	2,586	2,192	33	3
4	B204	177	11	105	355	17,234	86,711	245,508	3,050,498	144	2,645	2,146	33	3
5	B6933	162	9	105	265	18,909	124,001	215,007	3,063,365	155	2,657	2,189	33	3
6	B78^T^	150	7	105	339	20,421	154,115	419,336	3,063,182	187	2,655	2,232	33	3
7	B8044	248	14	125	307	12,224	71,122	191,659	3,031,756	88	2,729	2,192	32	3
8	G21	134	8	105	375	23,280	122,139	325,477	3,119,634	202	2,729	2,210	32	3
9	G44	209	11	105	395	14,412	98,426	237,547	3,012,109	192	2,589	2,194	33	3
10	FM88.90	124	6	119	702	24,150	170,197	303,455	2,994,627	214	2,581	2,179	33	3
11	NSW15	244	16	105	299	12,427	71,065	153,611	3,032,384	163	2,593	2,149	31	3
12	NSW5	255	11	105	270	11,912	75,956	380,118	3,037,586	154	2,605	2,367	33	3
13	Q17	213	13	105	258	14,230	80,605	249,233	3,031,122	272	2,599	2,161	33	3
14	ST190	125	7	105	334	24,530	155,235	321,663	3,066,306	181	2,688	2,468	33	3
15	ST195	151	7	105	313	20,461	148,017	390,887	3,089,721	140	2,697	2,280	33	3
16	ST210	141	7	105	496	21,714	151,573	470,264	3,061,741	182	2,659	2,200	33	3
17	ST265	309	14	105	444	10,103	57,007	342,883	3,122,061	174	2,717	2,276	33	3
18	Vic2	140	9	105	255	21,655	124,009	239,667	3,031,724	231	2,606	2,160	33	3
19	WA100	279	13	105	436	11,265	77,247	316,027	3,142,952	153	2,705	2,519	33	3

n = number of contigs, n:N50 = number of contigs in the N50, Min = minimum contig length, Max = Maximum contig length, Sum = sum of all contig lengths, Coverage = average read depth of coverage for contigs, Proteins = total number of predicted protein genes, Fl-gene = full length genes (not fragmented), tRNA = total number of transfer RNA genes, rRNA = total number of ribosomal RNA genes.

### Gene predictions

Annotation via RAST resulted in a broadly similar gene content, with gene numbers ranging from 2586 in strain NX to 2795 in strain FMV89.3323 (Table 2). The number of full-length genes identified at 90% reference WA1 gene coverage and percent sequence identity are reported in Table 2, and are discussed later.

### *B*. *hyodysenteriae* genome alignments

Although the 19 genome assemblies were fragmented, genome sequence alignment coverage was 99.5% at greater than 80% identity (and 95.2% at greater than 90% identity) to the reference strain WA1, except for strains FMV89.3323, ST265 and WA100. Overall, for the 19 strains the average percentage identity of aligned sequence was greater than 94.96% (Table 3). This allowed the direct investigation of the common gene content (core set) within the species, and genes present at a lower percentage that allowed cluster identity, or others that were unique to a strain (Table A in S1 Table). Across all strains there were 2,354 core genes (88%) and on average 255 ancillary genes (8.6%) and 77 unique genes (2.9%) per strain.

**Table 3:** *Brachyspira hyodysenteriae* strain genome sequence alignment length, average identity and unaligned sequence length to the reference WA1 genome NC_012225.

	Strain	Aligned length to reference (bp)	Average percent identity of aligned sequence to the reference	Unaligned sequence to the reference (bp)	Percentage unaligned sequence to the reference
1	865	2,996,613	95.81	6,128	0.20
2	FMV89.3323	2,988,495	95.76	79,435	2.59
3	NX	2,949,915	95.77	5,405	0.18
4	B204	2,988,672	96.04	2,655	0.09
5	B6933	3,010,745	95.85	3,260	0.11
6	B78^T^	2,992,496	95.48	11,269	0.38
7	B8044	2,976,881	96.15	9,537	0.32
8	G21	3,008,904	95.56	6,972	0.23
9	G44	2,994,105	96.23	4,778	0.16
10	NSW15	2,965,907	96.58	8,309	0.28
11	NSW5	3,071,900	96.90	5,550	0.18
12	Q17	3,085,456	97.28	4,070	0.13
13	ST190	3,021,359	95.83	3,015	0.10
14	ST195	2,987,300	95.79	11,150	0.37
15	ST210	3,025,920	96.19	3,235	0.11
16	ST265	2,963,498	96.19	55,467	1.84
17	Vic2	3,064,904	96.83	3,236	0.11
18	WA100	2,983,257	96.41	61,685	2.03
19	FM88.90	2,972,329	94.96	15,885	0.53

bp = base pair

Strain contigs that did not align to the reference ranged from 2,655 to 79,435 bases in length; this represented 0.09% to 2.59% of the assembled genome. Those strains with the higher percentages of non-aligned sequence consisted of mostly short fragmented sequences less than 500 bp. The unaligned strain sequences were investigated, and for 13 strains a total of 271 genes were extracted, ranging from 1 to 103 genes for a strain, with the remaining strains having no gene predictions in the unique sequence. Of the 271 genes, 215 (79%) were unknown hypothetical, 11 (4%) known hypothetical and 17 (6%) phage related genes (found in five strains). A total of 28 (10%) WA1 reference genes also were found in these regions, ranging from two to seven genes in 10 strains. Of these, putative methyl-accepting chemotaxis sensory transducer and methyltransferase2C FkbM were the most common, being found in eight strains (Table B in S1 Tables).

### Gene alignment to the reference WA1 genome

**Fig 1:**
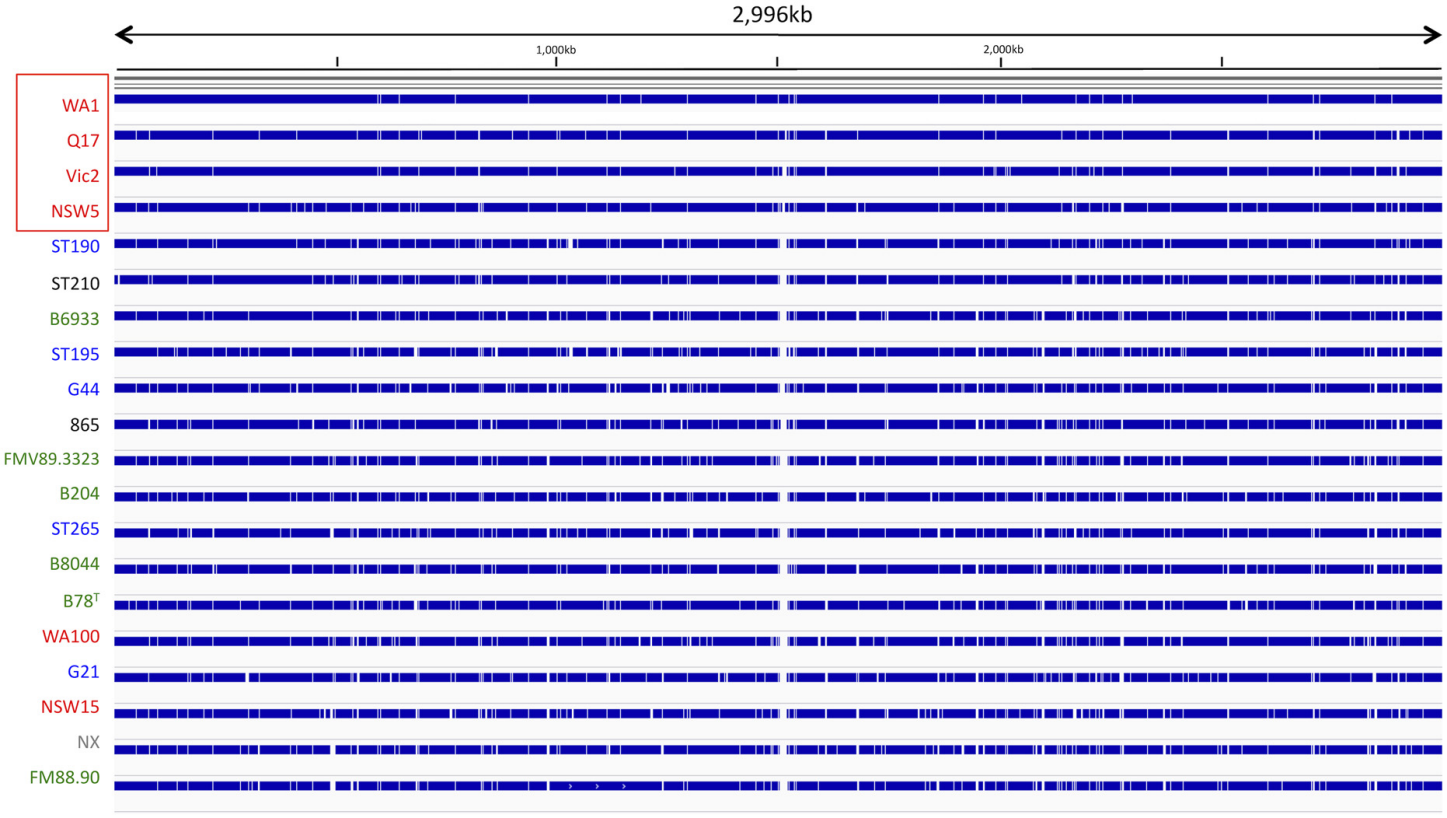
Gene alignments (shown in blue) of strains at better than 90% nucleotide sequence identity and 90% coverage to the reference NC_012225 3Mb genome. Strain labels are color-coded, Australia (red), Japan and Korea (black), Europe (blue), North America (green) and Unknown (grey). The sub-cluster of four Australian strains with high sequence identity is boxed.

**Fig 2:**
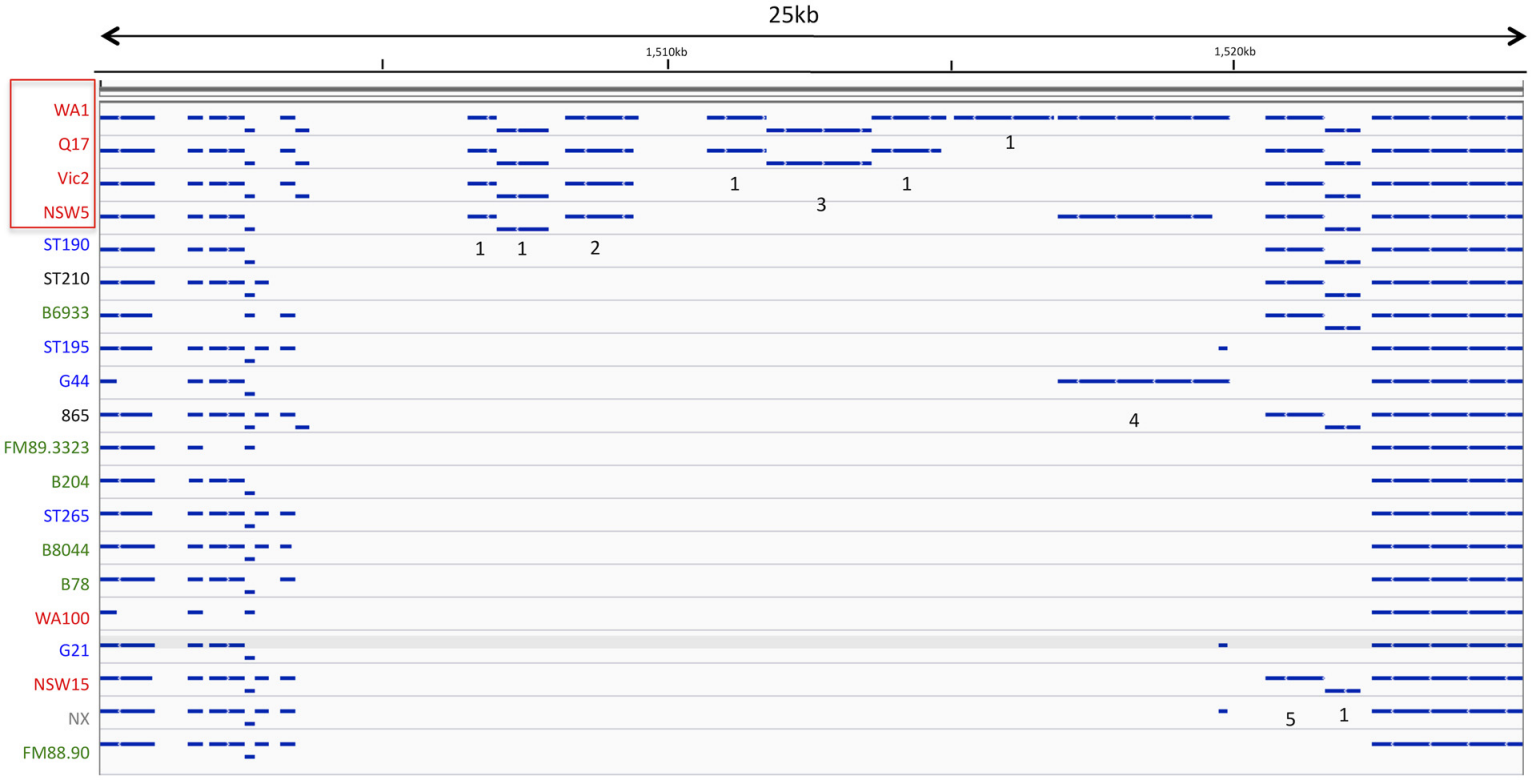
The 24 Kb gene alignment for a region (position 1,501 to 1,525 Kb in the NC_012225 reference genome), with five functional types of gene showing variable presence in the genome, 1) six hypotheticals, 2) mobile element protein, 3) bipolar DNA helicase HerA 4) type IIS restriction /modification enzyme, and 5) ADP-ribose 1"-phosphate phosphatase related protein.

The predicted strain gene alignment to the WA1 reference is shown in Fig 1. The closest strains by sequence identity to the WA1 reference were Queensland strain Q17 and New South Wales strain NSW5. No relatedness due to geographic location could be established. A region of approximately 24 Kb with variable gene alignments with the reference strain genes was identified at reference genomic position 1,501 Kb to 1,525 Kb. The gene content of this region included a type IIS restriction /modification enzyme, hypothetical proteins, a bipolar DNA helicase HerA, and a mobile element protein (Fig 2).

### Gene analysis

The number of full-length genes (90% sequence identity and coverage) for each strain was estimated based on the WA1 reference gene data set (Table 2). Strains NSW5, Q17 and Vic2 had greater than 98% of the gene calls full-length, and the remainder ranged from 92% for strain NSW15 up to 96% for strain ST190. In part this could be related to the fragmentation of the assemblies.

**Fig 3:**
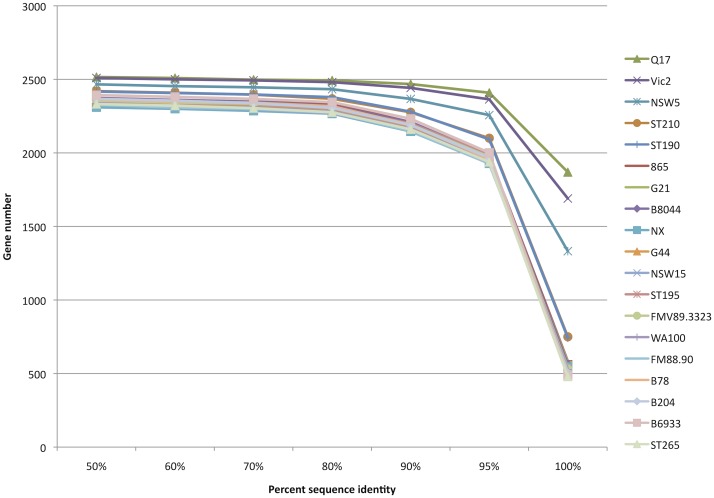
Number of genes of each strain identified aligning at ≥90% of the WA1 reference genes length at various percentage sequence alignment identity.

The distribution of full-length strain genes based on percent identity to the reference strain is shown in Fig 3. Strains Q17, Vic2 and NSW5 had the greatest number of genes with 100% sequence identity, being 1,869, 1,690 and 1,332 respectively, whilst the lowest number of 479 was found for strain ST265. The number of full-length genes for each strain grouped by percent identity to the reference is shown in Table C of S1 Tables.

### KEGG analysis

To determine any potential differences in metabolic pathways between strains, annotated enzymes for all gene predictions were extracted for each genome. Over a quarter of the genes could be assigned to a KEGG [31] enzyme (Table D in S1 Tables). A core set of 390 KEGG enzymes were in common to all the genomes and only a few enzymes were unique to a strain. These unique enzymes included an adenylate cyclase in NX, a retron-type RNA-directed DNA polymerase in B78^T^ and a restriction enzyme *Bcg*I beta subunit in NSW15. The remaining ten enzymes were all uniquely identified in B8044: Low-specificity L-threonine aldolase; dihydrodipicolinate reductase; two gene copies of dihydrodipicolinate synthas; beta-fructofuranosidase, N-acetylmuramic acid 6-phosphate etherase; three gene copies of cytidylate kinase;tRNA delta(2)-isopentenylpyrophosphate transferase; 3-deoxy-D-manno-octulosonic-acid transferase; Bis(5'-nucleosyl)-tetraphosphatase (asymmetrical); and an UDP-3-O-[3-hydroxymyristoyl] N-acetylglucosamine deacetylase. These unique enzymes are possible candidates from horizontal transfers.

### MLST phylogenetic analysis

**Fig 4:**
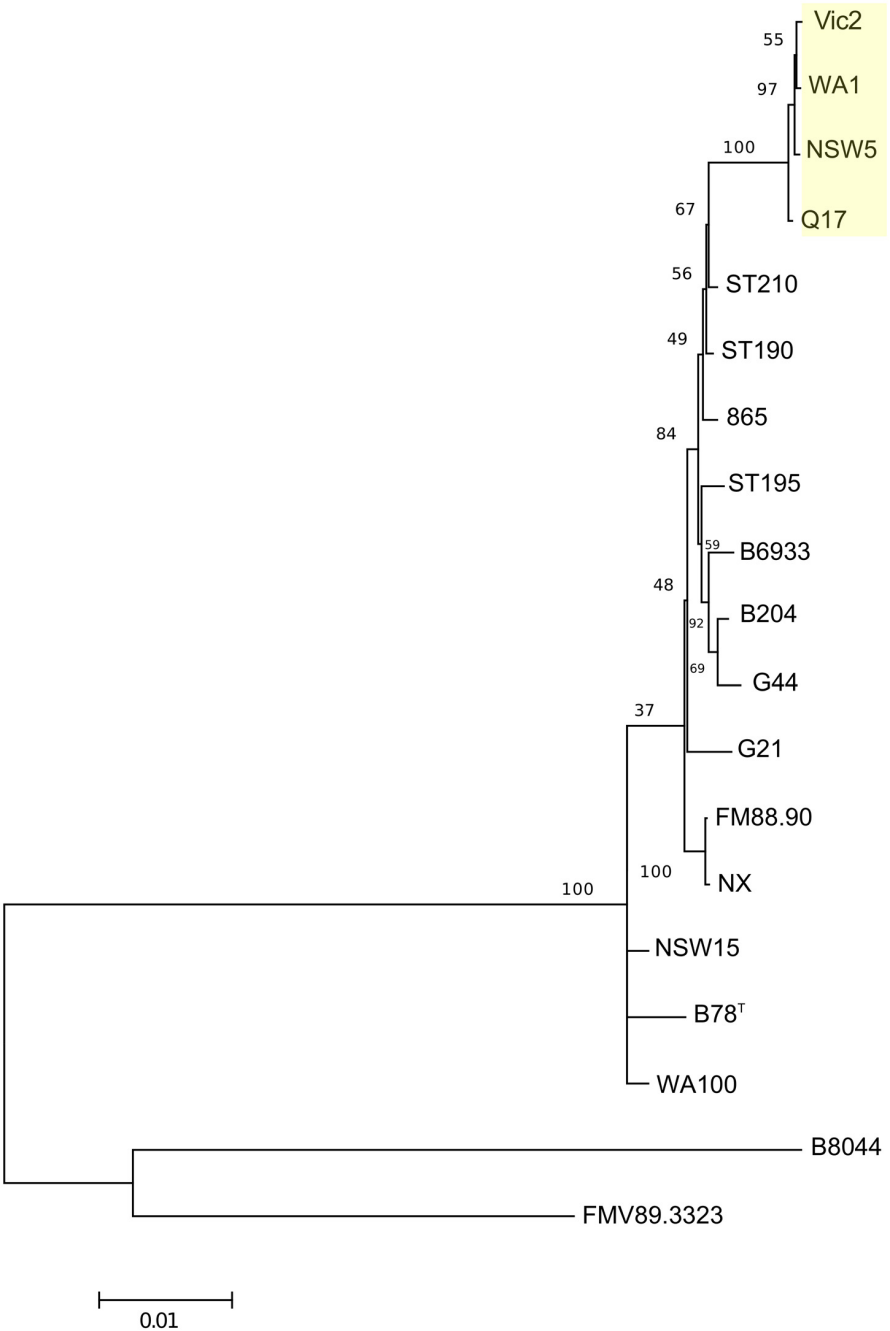
Bootstrap consensus Neighbour joining MLST phylogeny of 18 *B*. *hyodysenteriae* strains and the WA1 reference strain. The tree shows a sub-cluster (highlighted) of the Australia clade that includes strains WA1, NSW5, Vic2 and Q17. Numbers are the percentage of bootstrapped trees agreeing with the consensus. The distant outlier strain ST265 is not included.

There were two broad clusters in the phylogenetic tree (Fig 4), with one branch containing FMV89.3323 and B8044 and a second containing the other strains. Multiple sequence alignment showed that the major difference between this cluster and the other strains was in multiple variations in the *thi* gene (data not shown). This gene, encoding the thiamine biosynthesis protein, is important in synthesis of thiamine.

Within the larger cluster there was a sub-cluster of strains, which included a sub-cluster of some of the Australian strains including the reference WA1 genome, NSW5, Vic2 and Q17. This was in agreement with the pan genome alignment, and helps to support the validity of MLST as a method to estimate genetic relatedness of strains of *B*. *hyodysenteriae*. Unexpectedly, the *alp* gene of ST265 was found to be closely matched to that of a different species, *Brachyspira aalborgi*, itself an outlier amongst *Brachyspira* species and whose host is *Homo sapiens* (10). Taken on MLST alone, ST265 would not be classified in the species *B*. *hyodysenteriae*, however the clustering analysis in this paper suggests that it is. As this makes it a very distant outlier, it was not included in the *B*. *hyodysenteriae* MLST phylogeny.

### Proteome analysis

**Fig 5:**
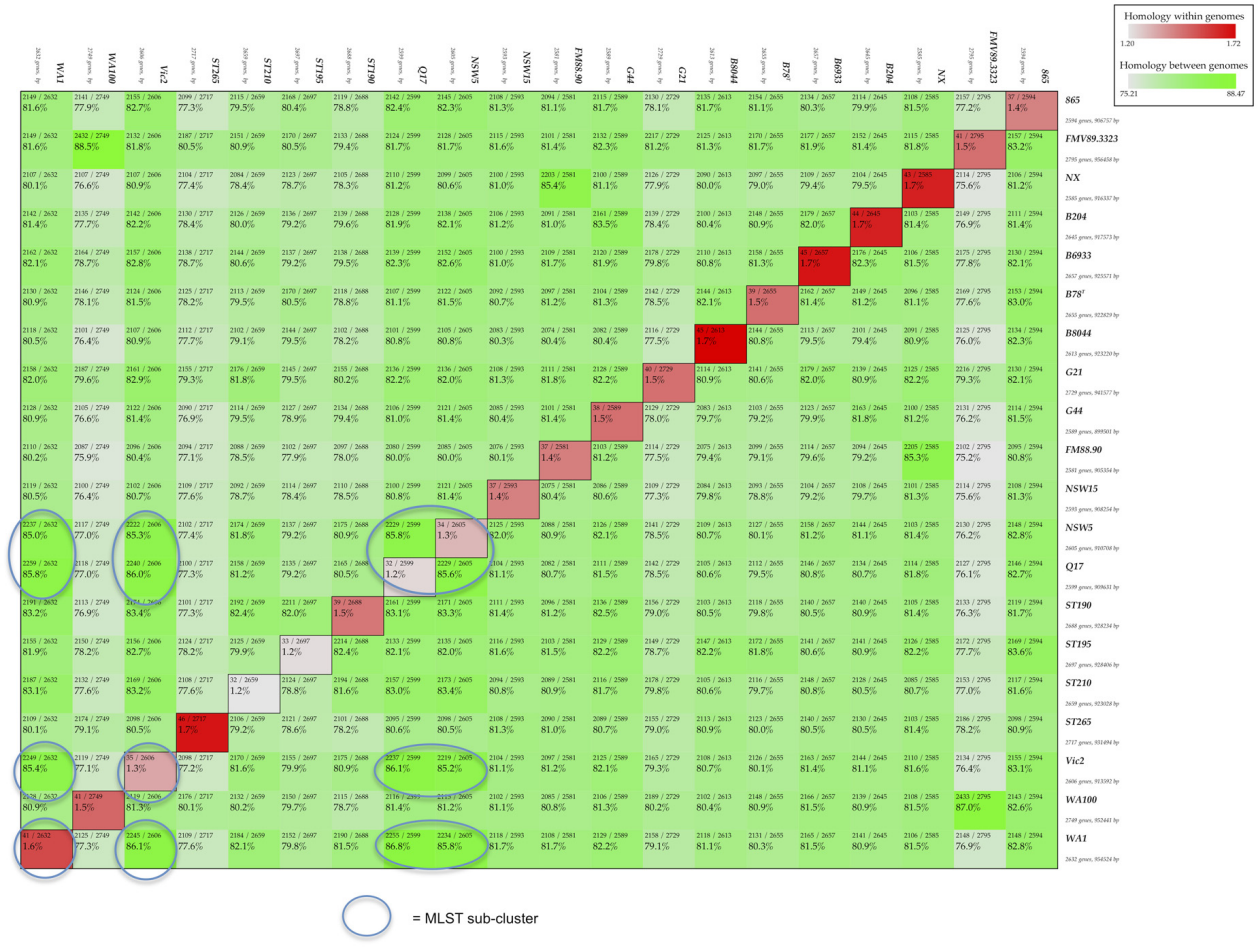
Protein similarity matrix showing 19 *B*. *hyodysenteriae* strains and the WA1 reference strain. Reading top to bottom, column by column, the matrix displays the percentage of homologous proteins between strains and on the diagonal the percentage of paralogous proteins (multiple copies of a protein) within the strain. Circled boxes represent the MLST sub-cluster of WA1, NSW5, Vic2 and Q17.

The protein matrix analysis of the 20 *B*. *hyodysenteriae* proteomes (19 new strains plus the reference WA1 proteome) largely mirrored the MLST results, but with some significant differences (Fig 5). One difference was the close clustering of ST265 with other *B*. *hyodysenteriae* strains despite the anomaly found in the MLST analysis. This supports the fact that ST265 is a strain of *B*. *hyodysenteriae* that has an *alp* gene that may have been horizontally transferred from another *Brachyspira* species. This is not a unique observation, as previously alleles of five other genes used in MLST have been deduced to have been transmitted between strains of *Brachyspira* species isolated from pigs [32].

In the protein matrix the strongest match to FMV89.3323 was with WA100, while in the MLST analysis the closest match was to B8044. By scanning down the B8044 column in Fig 5, it can be seen that the B8044 protein content most closely resembles that of ST195 (reading down the B8044 column) at 82.2% homology, rather than fellow MLST outlier FMV89.3323 with 81.3% homology. Scanning down the WA100 column in Fig 5, another curious result was found, with the strongest homology on the matrix being between WA100 and FMV89.3323 (88.5%), representing the strongest homology percentage of the matrix. Branching of the MLST tree suggests that WA100 is much more closely related to the other strains rather than to the MLST outliers FMV89.3323 and B8044.

There also were familiar patterns, with, for example, the sub-cluster defined in the MLST analysis of WA1, NSW5, Q17 and VIC2 being evident in the protein matrix analysis, with percentage of proteins that were homologous ranging from 85.7% to 87.3% in the new strains.

In summary, protein homology for all strains was relatively high, ranging from 75.6% to 88.5%, consistent with these strains all belonging to a single species (*B*. *hyodysenteriae*). This conclusion is further strengthened when compared to a similar multi-species protein matrix, calculated for a previous paper, which recorded protein homology percentages as low as 17% between different *Brachyspira* species, whilst between the three sequenced *B*. *pilosicoli* strains it varied from 54.9% to 68.4% (10).

### Virulence and pathogenesis

Putative genes involved with virulence and pathogenesis were identified in the genome of WA1 by Bellgard *et al*. [2]. Orthologous genes from the other 19 genomes were extracted for comparative purposes. In this method the tools BLAT, Muscle and Newick Utilities were used to produce FASTA databases, alignments and Neighbor-Joining trees. A basic count of the number in selected key genes believed to be important in virulence and pathogenesis was then completed.

Genes encoding proteins involved in host cell degradation, hemolysins and phospholipases, were very conserved with little variation between strains, with all seven hemolysins and all three phospholipases present in WA1 also being found in all the strains. In some cases the conservation was virtually 100%; for example, from a nucleotide MUSCLE alignment, it was shown that hemolysin B [33] was identical in all strains except for ST195 where there was only a synonymous one base substitution (base 668, A>C), while hemolysin C was identical in all strains (data not shown). The production of hemolysin has been considered a major virulence attribute of *B*. *hyodysenteriae* [5, 33]. This extreme conservation in a virulence attribute suggests that in order to target specific strains other markers are required.

There was more variation in two gene families associated with host cell adhesion, the lipoproteins and variable surface proteins. All 19 lipoproteins in WA1 were found in all the strains, whilst in contrast there was some variation in the number of VSPs. Most significantly, a revision of the reference WA1 strain showed that there were only four full-length variable surface proteins in the WA1 reference GenBank annotation, not nine as previously reported, probably due to miscounting in the original paper [2]. Strains 865, B204, B6933, B78^T^, B8044, NSW15, NSW5, Q17, ST190 and ST210 only had three of four VSPs, due to the absence of a full copy of *vspF*, suggesting a possible point of difference between strains.

There was little variation in gene content of motility genes amongst the strains. All strains had the same number and type of motility genes (*fla*, *fle*, *fli*, flagellar protein), suggesting that they had a shared motility phenotype. Chemotaxis genes showed more variation between strains. There was the same high number (30) of full-length *mcp* genes (methyl-accepting chemotaxis proteins), and this result strengthens the original finding that WA1 had twice the number of *mcp* genes found in other *Brachyspira* species [2].

**Fig 6:**
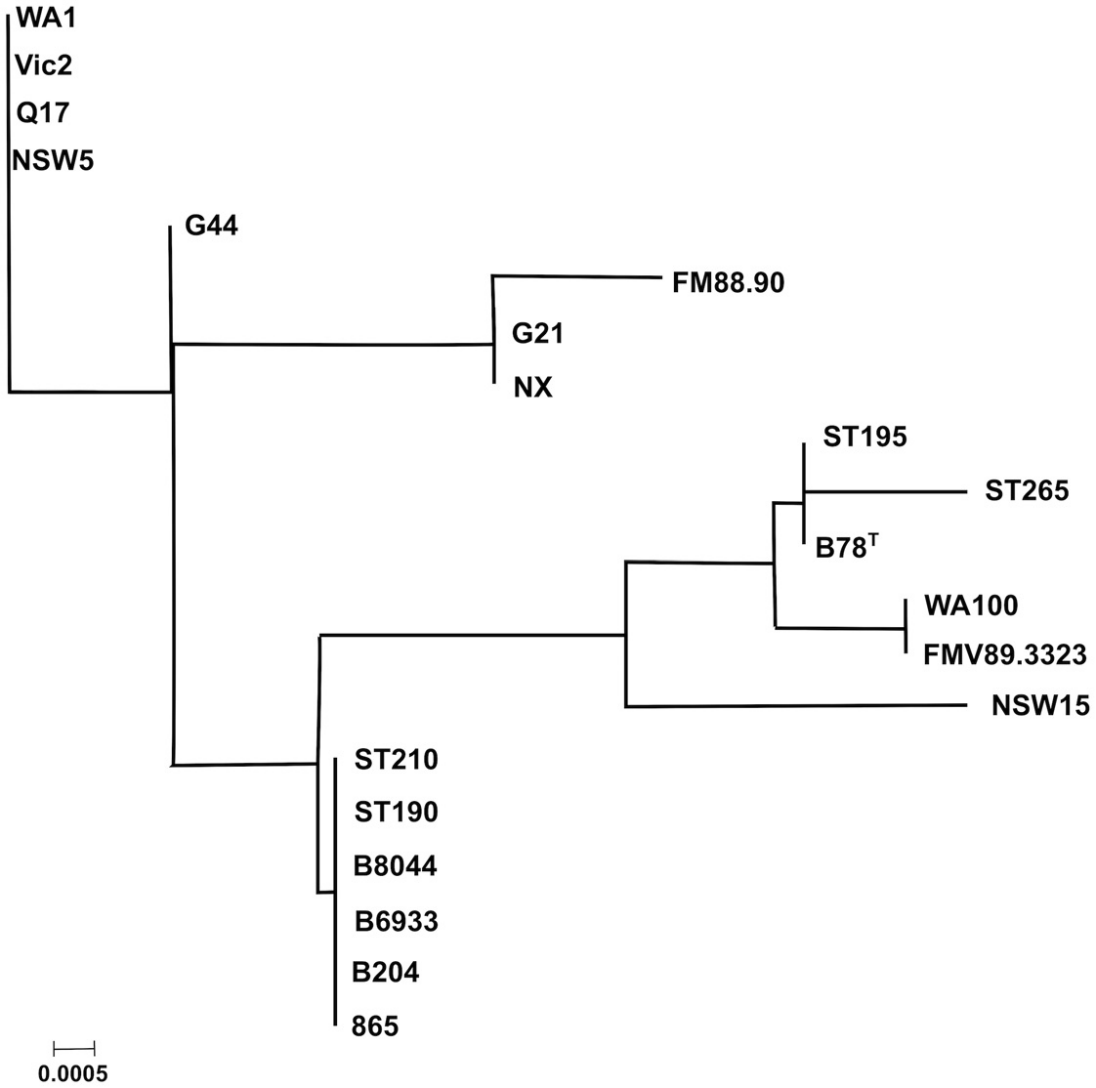
Cladogram of *che2* nucleotide sequences amongst the *B*. *hyodysenteriae* strains generated from a MUSCLE alignment.

As with WA1, 18 *che* (chemosensory transducer) genes were found in all strains (except for ST210 which had 17 genes, but there were some interesting sequence based differences). For example, there was distinct clustering in *cheA* (Fig 6). Firstly, like the MLST analysis, there was a sub-cluster of Australian strains WA1, Vic2, Q17 and NSW5. However, unlike in the MLST tree, FMV89.3323 *cheA* was closest to that of WA100, while B8044, ST190, ST210, ST204 and B6933 had identical *cheA* sequences. Further investigation of the translated protein sequences confirmed all variations were synonymous, therefore not rendering any possible functional changes (data not shown). This conclusion also raises the possibility of there being alternative codon biases within different strains.

Other gene families of note included those encoding ankyrin-like proteins, NADH oxidase (*nox*), peptidases, proteases, the *rfb* genes and glycosyl transferases (Table 4). Ankyrin proteins are known to bind to the host chromatin and could play a critical role in the interaction with the host cell [34]. There was some variation in the number of ankyrin-like proteins amongst the strains, but all had at least 48 full-length copies. All strains had the same high number of genes within the *nox*, peptidase and protease families. The proteases in particular are likely to be important, being involved in virulence via the destruction of host tissues.

**Table 4:** Number of full-length genes per strain with ≥ 90% identity to selected WA1 reference genes and gene families associated with virulence and pathogenicity.

Strain	Ankyrin-like protein	Nox	Peptidase	Protease	rfbDABC operon (Plasmid)	glycosyl transferase (Plasmid)
Reference WA1	52	2	41	18	4	6
865	52	2	41	18	4	6
FMV89.3323	48	2	41	17	4	6
NX	52	2	40	17	0	1
B204	52	2	41	18	4	9
B6933	51	2	41	18	4	6
B78^T^	52	2	41	18	4	5
B8044	52	2	41	18	4	5
G21	52	2	41	17	4	6
G44	52	2	40	18	2	5
FM88.90	52	2	41	17	0	1
NSW15	52	2	41	18	4	5
NSW5	52	2	40	18	4	5
Q17	52	2	40	18	4	5
ST190	52	2	40	18	4	6
ST195	52	2	41	18	4	4
ST210	52	2	41	18	4	6
ST265	49	2	41	18	4	5
Vic2	52	2	41	18	4	7
WA100	48	2	41	17	4	6

All the *B*. *hyodysenteriae* strains, except NX, had some evidence for a plasmid being present, as genes that were plasmid-encoded in WA1 were identified. An important plasmid region is the *rfbBADC* operon, with an arrangement unique to *B*. *hyodysenteriae*. It is predicted to be involved in lipooligosaccharide (LOS) biosynthesis, with LOS being thought to be important in virulence in *B*. *hyodysenteriae* [1]. CDSs matching to the WA1 plasmid *rfbBADC* operon were found in 17 of 19 non-WA1 strains, while no *rfb* genes were found in the incomplete genomes of NX and FM88.90 (Table 4). One of the matching strains, G44, only had complete *rfbA* and *rfbD* copies and a partial copy of *rfbB*. Further comparison to glycosyltransferases found in the WA1 plasmid showed a similar pattern with NX and FM88.90 only having one such gene, whilst the others had five or six (Table 4). This is strongly indicative of NX and FM88.90 lacking a plasmid matching the ~36 Kb plasmid of WA1. Lack of these plasmid genes potentially could reduce their virulence [35].

### Spirochete GenBank

All genomes have been deposited in DDBJ/EMBL/GenBank under BioProject PRJNA272555 (http://www.ncbi.nlm.nih.gov/bioproject/PRJNA272555/). This Whole Genome Shotgun project has been deposited at DDBJ/EMBL/GenBank under the accessions JXNA00000000-JXNS00000000. The version described in this paper is version JXNA01000000-JXNS01000000.

## Conclusion

At a genome level the 19 newly sequenced *B*. *hyodysenteriae* strains showed a high percent identity despite originating from widely different geographic locations. This is consistent with *B*. *hyodysenteriae* being a relatively conserved clonal species, and is in contrast to the highly recombinant species *B*. *pilosicoli* that also is pathogenic in pigs. Results from the genomic analysis support the use of MLST as a means to assess relatedness between *B*. *hyodysenteriae* strains. Despite the general conservation of the species, information that could be useful for future differential phenotypic studies of different *B*. *hyodysenteriae* strains was found. Other interesting results included evidence for a probable horizontal transfer of *alp* from a different *Brachyspira* species to *B*. *hyodysenteriae* ST265; the presence of a separate sub-cluster of some Australian *B*. *hyodysenteriae* strains; and a 100% conservation amongst some hemolysin genes which are considered to have a key role in the pathogenesis of *B*. *hyodysenteriae*.

All strains contained unique genes and had variance in gene sequence content in various important genes involved in virulence and/or pathogenesis that could confer phenotypic differences. There also was evidence that strains NX, FM88.90 and possibly G44 do not have a plasmid, at least one similar to the ~36 Kb plasmid found in reference genome WA1, and thus are likely to have marked phenotypic differences from other strains.

In summary, we have created an important repository of genomic information on a bacterial species about which there is still much that is unknown. We envisage that this study will advance the further refinement of these genomes as well as phenotypic investigation of *B*. *hyodysenteriae* virulence and pathogenesis.

## Supporting Information

S1 Tables Supporting gene content tables discussed in this study. (DOCX)
