# Supplementary material for: Analysis of Multiple Brachyspira hyodysenteriae Genomes Confirms That the Species Is Relatively Conserved but Has Potentially Important Strain Variation
Source: PLoS One. 2015 Jun 22;10(6):e0131050. doi: 10.1371/journal.pone.0131050 (PMC4476648; doi:10.1371/journal.pone.0131050)
Supplement: S1 Tables — (DOCX) [file pone.0131050.s001.docx]

Table A. Orthomcl  clustering of the *Brachyspira hyodysenteriae* isolates.

| Isolate | Total genes | Core | Ancillary | Unique |
| --- | --- | --- | --- | --- |
| 865 | 2,594 | 2,348 | 179 | 67 |
| FMV89.3323 | 2,795 | 2,361 | 317 | 117 |
| NX | 2,585 | 2,344 | 172 | 69 |
| B204 | 2,645 | 2,352 | 217 | 76 |
| B6933 | 2,657 | 2,351 | 234 | 72 |
| B78^T^ | 2,655 | 2,351 | 224 | 80 |
| B8044 | 2,613 | 2,359 | 186 | 68 |
| G21 | 2,729 | 2,347 | 291 | 91 |
| G44 | 2,589 | 2,347 | 167 | 75 |
| FM88.90 | 2,593 | 2,343 | 178 | 72 |
| NSW15 | 2,676 | 2,342 | 258 | 76 |
| NSW5 | 2,605 | 2,346 | 180 | 79 |
| Q17 | 2,599 | 2,351 | 181 | 67 |
| ST190 | 2,688 | 2,362 | 254 | 72 |
| ST195 | 2,697 | 2,353 | 271 | 73 |
| ST210 | 2,659 | 2,358 | 234 | 67 |
| ST265 | 2,717 | 2,349 | 280 | 88 |
| Vic2 | 2,606 | 2,343 | 194 | 69 |
| WA100 | 2,749 | 2,417 | 254 | 78 |
|  |  |  |  |  |
| Av. | 2,655 | 2,354 | 225 | 77 |

Table B. Gene content of all unaligned contigs

| Contig_gene | Source | Feature | start | end | score | strand | frame | Description |
| --- | --- | --- | --- | --- | --- | --- | --- | --- |
| NODE_108_length_1101_cov_138.847412 | FIG | CDS | 73 | 1014 | . | + | 1 | ID=fig\|159.4.peg.300;Name=Hypothetical protein Cj1433c |
| NODE_84_length_1658_cov_358.871521 | FIG | CDS | 39 | 296 | . | + | 0 | ID=fig\|159.4.peg.2560;Name=putative methyl-accepting chemotaxis sensory transducer |
| NODE_101_length_858_cov_176.363632 | FIG | CDS | 200 | 739 | . | + | 2 | ID=fig\|159.5.peg.91;Name=Phage protein |
| NODE_109_length_1080_cov_160.241669 | FIG | CDS | 74 | 1006 | . | + | 2 | ID=fig\|159.5.peg.264;Name=methyltransferase2C FkbM family domain protein |
| NODE_14_length_3264_cov_446.340393 | FIG | CDS | 66 | 320 | . | + | 0 | ID=fig\|159.5.peg.306;Name=hypothetical protein |
| NODE_14_length_3264_cov_446.340393 | FIG | CDS | 286 | 1287 | . | - | 1 | ID=fig\|159.5.peg.307;Name=hypothetical protein |
| NODE_14_length_3264_cov_446.340393 | FIG | CDS | 1713 | 1838 | . | + | 0 | ID=fig\|159.5.peg.308;Name=hypothetical protein |
| NODE_14_length_3264_cov_446.340393 | FIG | CDS | 2063 | 2608 | . | + | 2 | ID=fig\|159.5.peg.309;Name=hypothetical protein |
| NODE_14_length_3264_cov_446.340393 | FIG | CDS | 2605 | 2955 | . | + | 1 | ID=fig\|159.5.peg.310;Name=hypothetical protein |
| NODE_20_length_1569_cov_415.068848 | FIG | CDS | 375 | 656 | . | + | 0 | ID=fig\|159.5.peg.480;Name=hypothetical protein |
| NODE_20_length_1569_cov_415.068848 | FIG | CDS | 649 | 831 | . | + | 1 | ID=fig\|159.5.peg.481;Name=hypothetical protein |
| NODE_20_length_1569_cov_415.068848 | FIG | CDS | 816 | 1022 | . | + | 0 | ID=fig\|159.5.peg.482;Name=hypothetical protein |
| NODE_20_length_1569_cov_415.068848 | FIG | CDS | 1130 | 1414 | . | + | 2 | ID=fig\|159.5.peg.483;Name=hypothetical protein |
| NODE_26_length_3845_cov_456.821075 | FIG | CDS | 352 | 1002 | . | - | 1 | ID=fig\|159.5.peg.1063;Name=hypothetical protein |
| NODE_26_length_3845_cov_456.821075 | FIG | CDS | 1119 | 2696 | . | - | 0 | ID=fig\|159.5.peg.1064;Name=hypothetical protein |
| NODE_26_length_3845_cov_456.821075 | FIG | CDS | 2719 | 2883 | . | - | 1 | ID=fig\|159.5.peg.1065;Name=hypothetical protein |
| NODE_26_length_3845_cov_456.821075 | FIG | CDS | 2909 | 3139 | . | - | 2 | ID=fig\|159.5.peg.1066;Name=hypothetical protein |
| NODE_26_length_3845_cov_456.821075 | FIG | CDS | 3139 | 3348 | . | - | 1 | ID=fig\|159.5.peg.1067;Name=hypothetical protein |
| NODE_26_length_3845_cov_456.821075 | FIG | CDS | 3361 | 3663 | . | - | 1 | ID=fig\|159.5.peg.1068;Name=hypothetical protein |
| NODE_28_length_514_cov_235.052536 | FIG | CDS | 44 | 298 | . | - | 2 | ID=fig\|159.5.peg.1273;Name=hypothetical protein |
| NODE_57_length_4109_cov_469.722565 | FIG | CDS | 127 | 591 | . | - | 1 | ID=fig\|159.5.peg.2056;Name=hypothetical protein |
| NODE_57_length_4109_cov_469.722565 | FIG | CDS | 888 | 1070 | . | - | 0 | ID=fig\|159.5.peg.2057;Name=hypothetical protein |
| NODE_57_length_4109_cov_469.722565 | FIG | CDS | 1081 | 1269 | . | - | 1 | ID=fig\|159.5.peg.2058;Name=hypothetical protein |
| NODE_57_length_4109_cov_469.722565 | FIG | CDS | 1279 | 1950 | . | - | 1 | ID=fig\|159.5.peg.2059;Name=RNA polymerase sigma factor SigB |
| NODE_57_length_4109_cov_469.722565 | FIG | CDS | 1947 | 2135 | . | - | 0 | ID=fig\|159.5.peg.2060;Name=hypothetical protein |
| NODE_57_length_4109_cov_469.722565 | FIG | CDS | 2132 | 2641 | . | - | 2 | ID=fig\|159.5.peg.2061;Name=hypothetical protein |
| NODE_57_length_4109_cov_469.722565 | FIG | CDS | 2638 | 2859 | . | - | 1 | ID=fig\|159.5.peg.2062;Name=hypothetical protein |
| NODE_57_length_4109_cov_469.722565 | FIG | CDS | 2856 | 3017 | . | - | 0 | ID=fig\|159.5.peg.2063;Name=hypothetical protein |
| NODE_57_length_4109_cov_469.722565 | FIG | CDS | 3020 | 3376 | . | - | 2 | ID=fig\|159.5.peg.2064;Name=hypothetical protein |
| NODE_57_length_4109_cov_469.722565 | FIG | CDS | 3373 | 3789 | . | - | 1 | ID=fig\|159.5.peg.2065;Name=hypothetical protein |
| NODE_57_length_4109_cov_469.722565 | FIG | CDS | 3872 | 4087 | . | - | 2 | ID=fig\|159.5.peg.2066;Name=hypothetical protein |
| NODE_62_length_19261_cov_412.567108 | FIG | CDS | 344 | 1654 | . | + | 2 | ID=fig\|159.5.peg.2080;Name=massive surface protein MspG |
| NODE_62_length_19261_cov_412.567108 | FIG | CDS | 1672 | 2283 | . | + | 1 | ID=fig\|159.5.peg.2081;Name=hypothetical protein |
| NODE_62_length_19261_cov_412.567108 | FIG | CDS | 2300 | 3313 | . | + | 2 | ID=fig\|159.5.peg.2082;Name=hypothetical protein |
| NODE_62_length_19261_cov_412.567108 | FIG | CDS | 3329 | 3706 | . | + | 2 | ID=fig\|159.5.peg.2083;Name=hypothetical protein |
| NODE_62_length_19261_cov_412.567108 | FIG | CDS | 3707 | 4219 | . | + | 2 | ID=fig\|159.5.peg.2084;Name=hypothetical protein |
| NODE_62_length_19261_cov_412.567108 | FIG | CDS | 4219 | 4644 | . | + | 1 | ID=fig\|159.5.peg.2085;Name=hypothetical protein |
| NODE_62_length_19261_cov_412.567108 | FIG | CDS | 4656 | 6224 | . | + | 0 | ID=fig\|159.5.peg.2086;Name=hypothetical protein |
| NODE_62_length_19261_cov_412.567108 | FIG | CDS | 6227 | 6688 | . | - | 2 | ID=fig\|159.5.peg.2087;Name=hypothetical protein |
| NODE_62_length_19261_cov_412.567108 | FIG | CDS | 6685 | 7638 | . | - | 1 | ID=fig\|159.5.peg.2088;Name=hypothetical protein |
| NODE_62_length_19261_cov_412.567108 | FIG | CDS | 7805 | 8182 | . | - | 2 | ID=fig\|159.5.peg.2089;Name=hypothetical protein |
| NODE_62_length_19261_cov_412.567108 | FIG | CDS | 8257 | 10407 | . | + | 1 | ID=fig\|159.5.peg.2090;Name=Gene Transfer Agent terminase protein |
| NODE_62_length_19261_cov_412.567108 | FIG | CDS | 10512 | 11951 | . | + | 0 | ID=fig\|159.5.peg.2091;Name=FIG00437581: hypothetical protein |
| NODE_62_length_19261_cov_412.567108 | FIG | CDS | 12151 | 13026 | . | + | 1 | ID=fig\|159.5.peg.2092;Name=hypothetical protein |
| NODE_62_length_19261_cov_412.567108 | FIG | CDS | 13019 | 13189 | . | + | 2 | ID=fig\|159.5.peg.2093;Name=hypothetical protein |
| NODE_62_length_19261_cov_412.567108 | FIG | CDS | 13204 | 14139 | . | + | 1 | ID=fig\|159.5.peg.2094;Name=hypothetical protein |
| NODE_62_length_19261_cov_412.567108 | FIG | CDS | 14236 | 15651 | . | + | 1 | ID=fig\|159.5.peg.2095;Name=hypothetical protein |
| NODE_62_length_19261_cov_412.567108 | FIG | CDS | 15741 | 16196 | . | + | 0 | ID=fig\|159.5.peg.2096;Name=hypothetical protein |
| NODE_62_length_19261_cov_412.567108 | FIG | CDS | 16209 | 16772 | . | + | 0 | ID=fig\|159.5.peg.2097;Name=hypothetical protein |
| NODE_62_length_19261_cov_412.567108 | FIG | CDS | 16985 | 17572 | . | + | 2 | ID=fig\|159.5.peg.2098;Name=hypothetical protein |
| NODE_62_length_19261_cov_412.567108 | FIG | CDS | 17569 | 17889 | . | + | 1 | ID=fig\|159.5.peg.2099;Name=hypothetical protein |
| NODE_62_length_19261_cov_412.567108 | FIG | CDS | 18049 | 18279 | . | + | 1 | ID=fig\|159.5.peg.2100;Name=hypothetical protein |
| NODE_80_length_376_cov_257.821808 | FIG | CDS | 57 | 365 | . | + | 0 | ID=fig\|159.5.peg.2306;Name=hypothetical protein |
| NODE_87_length_303_cov_474.042908 | FIG | CDS | 52 | 354 | . | - | 1 | ID=fig\|159.5.peg.2371;Name=hypothetical protein |
| NODE_97_length_1656_cov_229.862320 | FIG | CDS | 64 | 447 | . | - | 1 | ID=fig\|159.5.peg.2735;Name=hypothetical protein |
| NODE_97_length_1656_cov_229.862320 | FIG | CDS | 440 | 763 | . | - | 2 | ID=fig\|159.5.peg.2736;Name=hypothetical protein |
| NODE_97_length_1656_cov_229.862320 | FIG | CDS | 750 | 1178 | . | - | 0 | ID=fig\|159.5.peg.2737;Name=hypothetical protein |
| NODE_97_length_1656_cov_229.862320 | FIG | CDS | 1181 | 1651 | . | - | 2 | ID=fig\|159.5.peg.2738;Name=hypothetical protein |
| NODE_9_length_36060_cov_290.773926 | FIG | CDS | 351 | 1373 | . | - | 0 | ID=fig\|159.5.peg.2749;Name=hypothetical protein |
| NODE_9_length_36060_cov_290.773926 | FIG | CDS | 1520 | 2143 | . | + | 2 | ID=fig\|159.5.peg.2750;Name=Phage endolysin |
| NODE_9_length_36060_cov_290.773926 | FIG | CDS | 2136 | 2969 | . | + | 0 | ID=fig\|159.5.peg.2751;Name=FIG00438273: hypothetical protein |
| NODE_9_length_36060_cov_290.773926 | FIG | CDS | 2950 | 3069 | . | + | 1 | ID=fig\|159.5.peg.2752;Name=hypothetical protein |
| NODE_9_length_36060_cov_290.773926 | FIG | CDS | 3070 | 3345 | . | + | 1 | ID=fig\|159.5.peg.2753;Name=Hol |
| NODE_9_length_36060_cov_290.773926 | FIG | CDS | 3467 | 3721 | . | + | 2 | ID=fig\|159.5.peg.2754;Name=hypothetical protein |
| NODE_9_length_36060_cov_290.773926 | FIG | CDS | 3729 | 3971 | . | + | 0 | ID=fig\|159.5.peg.2755;Name=hypothetical protein |
| NODE_9_length_36060_cov_290.773926 | FIG | CDS | 3961 | 4533 | . | + | 1 | ID=fig\|159.5.peg.2756;Name=hypothetical protein |
| NODE_9_length_36060_cov_290.773926 | FIG | CDS | 4536 | 6002 | . | + | 0 | ID=fig\|159.5.peg.2757;Name=Phage terminase2C large subunit |
| NODE_9_length_36060_cov_290.773926 | FIG | CDS | 6083 | 7684 | . | + | 2 | ID=fig\|159.5.peg.2758;Name=Mu-like prophage FluMu protein gp29 |
| NODE_9_length_36060_cov_290.773926 | FIG | CDS | 7684 | 9033 | . | + | 1 | ID=fig\|159.5.peg.2759;Name=Phage (Mu-like) virion morphogenesis protein |
| NODE_9_length_36060_cov_290.773926 | FIG | CDS | 9226 | 10290 | . | - | 1 | ID=fig\|159.5.peg.2760;Name=ankyrin repeat protein2C putative |
| NODE_9_length_36060_cov_290.773926 | FIG | CDS | 10340 | 10717 | . | - | 2 | ID=fig\|159.5.peg.2761;Name=hypothetical protein |
| NODE_9_length_36060_cov_290.773926 | FIG | CDS | 10758 | 11498 | . | - | 0 | ID=fig\|159.5.peg.2762;Name=hypothetical protein |
| NODE_9_length_36060_cov_290.773926 | FIG | CDS | 11660 | 11908 | . | + | 2 | ID=fig\|159.5.peg.2763;Name=hypothetical protein |
| NODE_9_length_36060_cov_290.773926 | FIG | CDS | 11937 | 12266 | . | + | 0 | ID=fig\|159.5.peg.2764;Name=hypothetical protein |
| NODE_9_length_36060_cov_290.773926 | FIG | CDS | 12288 | 12905 | . | + | 0 | ID=fig\|159.5.peg.2765;Name=hypothetical protein |
| NODE_9_length_36060_cov_290.773926 | FIG | CDS | 12934 | 13083 | . | + | 1 | ID=fig\|159.5.peg.2766;Name=hypothetical protein |
| NODE_9_length_36060_cov_290.773926 | FIG | CDS | 13225 | 13656 | . | + | 1 | ID=fig\|159.5.peg.2767;Name=hypothetical protein |
| NODE_9_length_36060_cov_290.773926 | FIG | CDS | 13670 | 13954 | . | + | 2 | ID=fig\|159.5.peg.2768;Name=hypothetical protein |
| NODE_9_length_36060_cov_290.773926 | FIG | CDS | 14056 | 14457 | . | + | 1 | ID=fig\|159.5.peg.2769;Name=hypothetical protein |
| NODE_9_length_36060_cov_290.773926 | FIG | CDS | 14458 | 15387 | . | + | 1 | ID=fig\|159.5.peg.2770;Name=Phage protein |
| NODE_9_length_36060_cov_290.773926 | FIG | CDS | 15536 | 17263 | . | + | 2 | ID=fig\|159.5.peg.2771;Name=Phage transposase |
| NODE_9_length_36060_cov_290.773926 | FIG | CDS | 17467 | 18327 | . | + | 1 | ID=fig\|159.5.peg.2772;Name=Eha |
| NODE_9_length_36060_cov_290.773926 | FIG | CDS | 18356 | 18694 | . | + | 2 | ID=fig\|159.5.peg.2773;Name=hypothetical protein |
| NODE_9_length_36060_cov_290.773926 | FIG | CDS | 18832 | 19326 | . | + | 1 | ID=fig\|159.5.peg.2774;Name=hypothetical protein |
| NODE_9_length_36060_cov_290.773926 | FIG | CDS | 19441 | 19656 | . | + | 1 | ID=fig\|159.5.peg.2775;Name=hypothetical protein |
| NODE_9_length_36060_cov_290.773926 | FIG | CDS | 19653 | 20123 | . | + | 0 | ID=fig\|159.5.peg.2776;Name=hypothetical protein |
| NODE_9_length_36060_cov_290.773926 | FIG | CDS | 20295 | 21296 | . | + | 0 | ID=fig\|159.5.peg.2777;Name=Mu-like prophage FluMu I protein |
| NODE_9_length_36060_cov_290.773926 | FIG | CDS | 21333 | 22244 | . | + | 0 | ID=fig\|159.5.peg.2778;Name=Phage major capsid protein |
| NODE_9_length_36060_cov_290.773926 | FIG | CDS | 22263 | 22574 | . | + | 0 | ID=fig\|159.5.peg.2779;Name=hypothetical protein |
| NODE_9_length_36060_cov_290.773926 | FIG | CDS | 22699 | 23136 | . | + | 1 | ID=fig\|159.5.peg.2780;Name=hypothetical protein |
| NODE_9_length_36060_cov_290.773926 | FIG | CDS | 23139 | 23603 | . | + | 0 | ID=fig\|159.5.peg.2781;Name=hypothetical protein |
| NODE_9_length_36060_cov_290.773926 | FIG | CDS | 23626 | 24159 | . | + | 1 | ID=fig\|159.5.peg.2782;Name=hypothetical protein |
| NODE_9_length_36060_cov_290.773926 | FIG | CDS | 24218 | 25714 | . | + | 2 | ID=fig\|159.5.peg.2783;Name=Bacteriophage tail sheath protein |
| NODE_9_length_36060_cov_290.773926 | FIG | CDS | 25758 | 26111 | . | + | 0 | ID=fig\|159.5.peg.2784;Name=hypothetical protein |
| NODE_9_length_36060_cov_290.773926 | FIG | CDS | 26131 | 26424 | . | + | 1 | ID=fig\|159.5.peg.2785;Name=hypothetical protein |
| NODE_9_length_36060_cov_290.773926 | FIG | CDS | 26489 | 26608 | . | + | 2 | ID=fig\|159.5.peg.2786;Name=hypothetical protein |
| NODE_9_length_36060_cov_290.773926 | FIG | CDS | 26601 | 28607 | . | + | 0 | ID=fig\|159.5.peg.2787;Name=Phage tail length tape-measure protein |
| NODE_9_length_36060_cov_290.773926 | FIG | CDS | 28648 | 29940 | . | + | 1 | ID=fig\|159.5.peg.2788;Name=hypothetical protein |
| NODE_9_length_36060_cov_290.773926 | FIG | CDS | 29958 | 30968 | . | + | 0 | ID=fig\|159.5.peg.2789;Name=hypothetical protein |
| NODE_9_length_36060_cov_290.773926 | FIG | CDS | 30972 | 31538 | . | + | 0 | ID=fig\|159.5.peg.2790;Name=hypothetical protein |
| NODE_9_length_36060_cov_290.773926 | FIG | CDS | 31540 | 31863 | . | + | 1 | ID=fig\|159.5.peg.2791;Name=hypothetical protein |
| NODE_9_length_36060_cov_290.773926 | FIG | CDS | 31856 | 32902 | . | + | 2 | ID=fig\|159.5.peg.2792;Name=Phage FluMu protein gp47 |
| NODE_9_length_36060_cov_290.773926 | FIG | CDS | 32893 | 33459 | . | + | 1 | ID=fig\|159.5.peg.2793;Name=hypothetical protein |
| NODE_9_length_36060_cov_290.773926 | FIG | CDS | 33474 | 34913 | . | + | 0 | ID=fig\|159.5.peg.2794;Name=hypothetical protein |
| NODE_9_length_36060_cov_290.773926 | FIG | CDS | 35384 | 35983 | . | + | 2 | ID=fig\|159.5.peg.2795;Name=hypothetical protein |
| NODE_52_length_2375_cov_186.602951 | FIG | CDS | 435 | 614 | . | + | 0 | ID=fig\|159.6.peg.2143;Name=hypothetical protein |
| NODE_52_length_2375_cov_186.602951 | FIG | CDS | 1300 | 2382 | . | + | 1 | ID=fig\|159.6.peg.2144;Name=hypothetical protein |
| NODE_14_length_858_cov_186.367126 | FIG | CDS | 172 | 711 | . | - | 1 | ID=fig\|159.11.peg.590;Name=Phage protein |
| NODE_28_length_2289_cov_310.463074 | FIG | CDS | 150 | 1169 | . | + | 0 | ID=fig\|159.11.peg.1254;Name=putative mRNA-binding protein |
| NODE_40_length_2390_cov_205.307114 | FIG | CDS | 720 | 1172 | . | - | 0 | ID=fig\|159.11.peg.1516;Name=hypothetical protein |
| NODE_40_length_2390_cov_205.307114 | FIG | CDS | 1667 | 2353 | . | - | 2 | ID=fig\|159.11.peg.1517;Name=Putative type IIS restriction /modification enzyme2C N-terminal half |
| NODE_40_length_2390_cov_205.307114 | FIG | CDS | 1172 | 1663 | . | - | 2 | ID=fig\|159.11.peg.1517;Name=Putative type IIS restriction /modification enzyme2C N-terminal half |
| NODE_88_length_1079_cov_162.861908 | FIG | CDS | 74 | 1006 | . | + | 2 | ID=fig\|159.11.peg.2563;Name=methyltransferase2C FkbM family domain protein |
| NODE_144_length_2375_cov_50.601685 | FIG | CDS | 674 | 1756 | . | + | 2 | ID=fig\|159.27.peg.317;Name=hypothetical protein |
| NODE_144_length_2375_cov_50.601685 | FIG | CDS | 2184 | 2363 | . | + | 0 | ID=fig\|159.27.peg.318;Name=hypothetical protein |
| NODE_66_length_1124_cov_80.473312 | FIG | CDS | 10 | 129 | . | + | 1 | ID=fig\|159.27.peg.2253;Name=hypothetical protein |
| NODE_66_length_1124_cov_80.473312 | FIG | CDS | 165 | 1106 | . | - | 0 | ID=fig\|159.27.peg.2254;Name=conserved hypothetical protein |
| NODE_87_length_1129_cov_77.809563 | FIG | CDS | 6 | 158 | . | + | 0 | ID=fig\|159.27.peg.2426;Name=hypothetical protein |
| NODE_87_length_1129_cov_77.809563 | FIG | CDS | 133 | 1167 | . | - | 1 | ID=fig\|159.27.peg.2427;Name=hypothetical protein |
| NODE_90_length_1129_cov_81.607620 | FIG | CDS | 134 | 1033 | . | + | 2 | ID=fig\|159.27.peg.2490;Name=hypothetical protein |
| NODE_10_length_2365_cov_180.770828 | FIG | CDS | 229 | 1128 | . | + | 1 | ID=fig\|159.12.peg.195;Name=hypothetical protein |
| NODE_10_length_2365_cov_180.770828 | FIG | CDS | 1464 | 1649 | . | + | 0 | ID=fig\|159.12.peg.196;Name=hypothetical protein |
| NODE_30_length_1108_cov_167.104691 | FIG | CDS | 112 | 1146 | . | - | 1 | ID=fig\|159.12.peg.1912;Name=methyltransferase2C FkbM family domain protein |
| NODE_85_length_1099_cov_176.317566 | FIG | CDS | 140 | 1081 | . | - | 2 | ID=fig\|159.12.peg.2655;Name=Hypothetical protein Cj1433c |
| NODE_73_length_1040_cov_387.094238 | FIG | CDS | 197 | 1021 | . | + | 2 | ID=fig\|159.13.peg.2326;Name=Gll4302 protein |
| NODE_12_length_2379_cov_13272.401367 | FIG | CDS | 46 | 483 | . | + | 1 | ID=fig\|159.15.peg.245;Name=hypothetical protein |
| NODE_12_length_2379_cov_13272.401367 | FIG | CDS | 564 | 1130 | . | + | 0 | ID=fig\|159.15.peg.246;Name=hypothetical protein |
| NODE_12_length_2379_cov_13272.401367 | FIG | CDS | 1558 | 1674 | . | + | 1 | ID=fig\|159.15.peg.247;Name=hypothetical protein |
| NODE_153_length_1116_cov_122.198029 | FIG | CDS | 155 | 1096 | . | - | 2 | ID=fig\|159.15.peg.438;Name=Hypothetical protein Cj1433c |
| NODE_48_length_1676_cov_361.362183 | FIG | CDS | 1433 | 1690 | . | - | 2 | ID=fig\|159.14.peg.1920;Name=putative methyl-accepting chemotaxis sensory transducer |
| NODE_58_length_1676_cov_422.945099 | FIG | CDS | 1433 | 1690 | . | - | 2 | ID=fig\|159.16.peg.1881;Name=putative methyl-accepting chemotaxis sensory transducer |
| NODE_49_length_2315_cov_164.664368 | FIG | CDS | 720 | 1151 | . | - | 0 | ID=fig\|159.18.peg.2268;Name=hypothetical protein |
| NODE_49_length_2315_cov_164.664368 | FIG | CDS | 1646 | 2332 | . | - | 2 | ID=fig\|159.18.peg.2269;Name=putative type IIS restriction/modification enzyme |
| NODE_49_length_2315_cov_164.664368 | FIG | CDS | 1151 | 1642 | . | - | 2 | ID=fig\|159.18.peg.2269;Name=putative type IIS restriction/modification enzyme |
| NODE_59_length_1259_cov_130.984909 | FIG | CDS | 126 | 1160 | . | - | 0 | ID=fig\|159.18.peg.2404;Name=methyltransferase2C FkbM family domain protein |
| NODE_73_length_1100_cov_129.781815 | FIG | CDS | 146 | 307 | . | - | 2 | ID=fig\|159.18.peg.2412;Name=hypothetical protein |
| NODE_73_length_1100_cov_129.781815 | FIG | CDS | 414 | 953 | . | - | 0 | ID=fig\|159.18.peg.2413;Name=Phage protein |
| NODE_9_length_3256_cov_1797.493530 | FIG | CDS | 382 | 1602 | . | - | 1 | ID=fig\|159.18.peg.2696;Name=FIG00438247: hypothetical protein |
| NODE_9_length_3256_cov_1797.493530 | FIG | CDS | 2931 | 3173 | . | - | 0 | ID=fig\|159.18.peg.2697;Name=hypothetical protein |
| NODE_119_length_760_cov_156.913162 | FIG | CDS | 124 | 771 | . | - | 1 | ID=fig\|159.20.peg.292;Name=hypothetical protein |
| NODE_127_length_1459_cov_154.736801 | FIG | CDS | 684 | 1385 | . | + | 0 | ID=fig\|159.20.peg.302;Name=hypothetical protein |
| NODE_129_length_1019_cov_343.630035 | FIG | CDS | 132 | 320 | . | + | 0 | ID=fig\|159.20.peg.311;Name=hypothetical protein |
| NODE_129_length_1019_cov_343.630035 | FIG | CDS | 407 | 646 | . | + | 2 | ID=fig\|159.20.peg.312;Name=hypothetical protein |
| NODE_129_length_1019_cov_343.630035 | FIG | CDS | 646 | 837 | . | + | 1 | ID=fig\|159.20.peg.313;Name=hypothetical protein |
| NODE_129_length_1019_cov_343.630035 | FIG | CDS | 839 | 976 | . | + | 2 | ID=fig\|159.20.peg.314;Name=hypothetical protein |
| NODE_136_length_13004_cov_147.587357 | FIG | CDS | 43 | 1452 | . | - | 1 | ID=fig\|159.20.peg.645;Name=FIG00437581: hypothetical protein |
| NODE_136_length_13004_cov_147.587357 | FIG | CDS | 1449 | 3590 | . | - | 0 | ID=fig\|159.20.peg.646;Name=Gene Transfer Agent terminase protein |
| NODE_136_length_13004_cov_147.587357 | FIG | CDS | 3786 | 4289 | . | + | 0 | ID=fig\|159.20.peg.647;Name=hypothetical protein |
| NODE_136_length_13004_cov_147.587357 | FIG | CDS | 4366 | 4944 | . | + | 1 | ID=fig\|159.20.peg.648;Name=hypothetical protein |
| NODE_136_length_13004_cov_147.587357 | FIG | CDS | 4956 | 5396 | . | + | 0 | ID=fig\|159.20.peg.649;Name=hypothetical protein |
| NODE_136_length_13004_cov_147.587357 | FIG | CDS | 5401 | 7338 | . | - | 1 | ID=fig\|159.20.peg.650;Name=hypothetical protein |
| NODE_136_length_13004_cov_147.587357 | FIG | CDS | 7354 | 7779 | . | - | 1 | ID=fig\|159.20.peg.651;Name=hypothetical protein |
| NODE_136_length_13004_cov_147.587357 | FIG | CDS | 7779 | 8291 | . | - | 0 | ID=fig\|159.20.peg.652;Name=hypothetical protein |
| NODE_136_length_13004_cov_147.587357 | FIG | CDS | 8292 | 8669 | . | - | 0 | ID=fig\|159.20.peg.653;Name=hypothetical protein |
| NODE_136_length_13004_cov_147.587357 | FIG | CDS | 8684 | 9697 | . | - | 2 | ID=fig\|159.20.peg.654;Name=hypothetical protein |
| NODE_136_length_13004_cov_147.587357 | FIG | CDS | 9714 | 10325 | . | - | 0 | ID=fig\|159.20.peg.655;Name=hypothetical protein |
| NODE_136_length_13004_cov_147.587357 | FIG | CDS | 10343 | 11644 | . | - | 2 | ID=fig\|159.20.peg.656;Name=hypothetical protein |
| NODE_136_length_13004_cov_147.587357 | FIG | CDS | 11729 | 12139 | . | - | 2 | ID=fig\|159.20.peg.657;Name=hypothetical protein |
| NODE_136_length_13004_cov_147.587357 | FIG | CDS | 12639 | 12911 | . | - | 0 | ID=fig\|159.20.peg.658;Name=hypothetical protein |
| NODE_182_length_580_cov_215.008621 | FIG | CDS | 347 | 574 | . | - | 2 | ID=fig\|159.20.peg.937;Name=hypothetical protein |
| NODE_190_length_832_cov_163.947113 | FIG | CDS | 71 | 424 | . | + | 2 | ID=fig\|159.20.peg.941;Name=hypothetical protein |
| NODE_208_length_840_cov_372.388092 | FIG | CDS | 118 | 555 | . | + | 1 | ID=fig\|159.20.peg.1071;Name=hypothetical protein |
| NODE_240_length_2294_cov_374.073669 | FIG | CDS | 1370 | 1534 | . | - | 2 | ID=fig\|159.20.peg.1243;Name=hypothetical protein |
| NODE_240_length_2294_cov_374.073669 | FIG | CDS | 1560 | 1793 | . | - | 0 | ID=fig\|159.20.peg.1244;Name=hypothetical protein |
| NODE_240_length_2294_cov_374.073669 | FIG | CDS | 1806 | 2108 | . | - | 0 | ID=fig\|159.20.peg.1245;Name=hypothetical protein |
| NODE_285_length_1116_cov_153.056458 | FIG | CDS | 155 | 1096 | . | - | 2 | ID=fig\|159.20.peg.1273;Name=Hypothetical protein Cj1433c |
| NODE_337_length_523_cov_403.193115 | FIG | CDS | 118 | 435 | . | + | 1 | ID=fig\|159.20.peg.1477;Name=hypothetical protein |
| NODE_342_length_839_cov_322.851013 | FIG | CDS | 372 | 653 | . | + | 0 | ID=fig\|159.20.peg.1501;Name=hypothetical protein |
| NODE_342_length_839_cov_322.851013 | FIG | CDS | 646 | 828 | . | + | 1 | ID=fig\|159.20.peg.1502;Name=hypothetical protein |
| NODE_56_length_13462_cov_151.044800 | FIG | CDS | 107 | 982 | . | + | 2 | ID=fig\|159.20.peg.1871;Name=hypothetical protein |
| NODE_56_length_13462_cov_151.044800 | FIG | CDS | 1186 | 1917 | . | + | 1 | ID=fig\|159.20.peg.1872;Name=hypothetical protein |
| NODE_56_length_13462_cov_151.044800 | FIG | CDS | 1910 | 3325 | . | + | 2 | ID=fig\|159.20.peg.1873;Name=hypothetical protein |
| NODE_56_length_13462_cov_151.044800 | FIG | CDS | 3413 | 3868 | . | + | 2 | ID=fig\|159.20.peg.1874;Name=hypothetical protein |
| NODE_56_length_13462_cov_151.044800 | FIG | CDS | 3881 | 4444 | . | + | 2 | ID=fig\|159.20.peg.1875;Name=hypothetical protein |
| NODE_56_length_13462_cov_151.044800 | FIG | CDS | 4657 | 5244 | . | + | 1 | ID=fig\|159.20.peg.1876;Name=hypothetical protein |
| NODE_56_length_13462_cov_151.044800 | FIG | CDS | 5241 | 5561 | . | + | 0 | ID=fig\|159.20.peg.1877;Name=hypothetical protein |
| NODE_56_length_13462_cov_151.044800 | FIG | CDS | 5871 | 6068 | . | + | 0 | ID=fig\|159.20.peg.1878;Name=hypothetical protein |
| NODE_56_length_13462_cov_151.044800 | FIG | CDS | 6085 | 6351 | . | + | 1 | ID=fig\|159.20.peg.1879;Name=hypothetical protein |
| NODE_56_length_13462_cov_151.044800 | FIG | CDS | 6542 | 7540 | . | + | 2 | ID=fig\|159.20.peg.1880;Name=hypothetical protein |
| NODE_56_length_13462_cov_151.044800 | FIG | CDS | 7533 | 8642 | . | - | 0 | ID=fig\|159.20.peg.1881;Name=hypothetical protein |
| NODE_56_length_13462_cov_151.044800 | FIG | CDS | 8647 | 9120 | . | - | 1 | ID=fig\|159.20.peg.1882;Name=hypothetical protein |
| NODE_56_length_13462_cov_151.044800 | FIG | CDS | 9532 | 10224 | . | + | 1 | ID=fig\|159.20.peg.1883;Name=hypothetical protein |
| NODE_56_length_13462_cov_151.044800 | FIG | CDS | 10221 | 10586 | . | + | 0 | ID=fig\|159.20.peg.1884;Name=hypothetical protein |
| NODE_56_length_13462_cov_151.044800 | FIG | CDS | 10589 | 10930 | . | + | 2 | ID=fig\|159.20.peg.1885;Name=hypothetical protein |
| NODE_56_length_13462_cov_151.044800 | FIG | CDS | 10936 | 12153 | . | + | 1 | ID=fig\|159.20.peg.1886;Name=hypothetical protein |
| NODE_56_length_13462_cov_151.044800 | FIG | CDS | 12150 | 12761 | . | + | 0 | ID=fig\|159.20.peg.1887;Name=hypothetical protein |
| NODE_63_length_4378_cov_369.139801 | FIG | CDS | 81 | 953 | . | + | 0 | ID=fig\|159.20.peg.2012;Name=FIG00438273: hypothetical protein |
| NODE_63_length_4378_cov_369.139801 | FIG | CDS | 953 | 1210 | . | + | 2 | ID=fig\|159.20.peg.2013;Name=hypothetical protein |
| NODE_63_length_4378_cov_369.139801 | FIG | CDS | 1194 | 1445 | . | + | 0 | ID=fig\|159.20.peg.2014;Name=hypothetical protein |
| NODE_63_length_4378_cov_369.139801 | FIG | CDS | 1436 | 1819 | . | + | 2 | ID=fig\|159.20.peg.2015;Name=hypothetical protein |
| NODE_63_length_4378_cov_369.139801 | FIG | CDS | 1919 | 2104 | . | + | 2 | ID=fig\|159.20.peg.2016;Name=hypothetical protein |
| NODE_63_length_4378_cov_369.139801 | FIG | CDS | 2236 | 2592 | . | + | 1 | ID=fig\|159.20.peg.2017;Name=Phage chromosome segregation protein |
| NODE_63_length_4378_cov_369.139801 | FIG | CDS | 2604 | 2828 | . | + | 0 | ID=fig\|159.20.peg.2018;Name=hypothetical protein |
| NODE_63_length_4378_cov_369.139801 | FIG | CDS | 2977 | 3678 | . | - | 1 | ID=fig\|159.20.peg.2019;Name=Integrase |
| NODE_86_length_222_cov_217.536041 | FIG | CDS | 72 | 233 | . | - | 0 | ID=fig\|159.20.peg.2428;Name=hypothetical protein |
| NODE_95_length_519_cov_204.483627 | FIG | CDS | 166 | 378 | . | + | 1 | ID=fig\|159.20.peg.2631;Name=hypothetical protein |
| NODE_98_length_383_cov_390.049622 | FIG | CDS | 229 | 432 | . | - | 1 | ID=fig\|159.20.peg.2632;Name=hypothetical protein |
| NODE_100_length_376_cov_290.114349 | FIG | CDS | 57 | 365 | . | + | 0 | ID=fig\|159.23.peg.1;Name=hypothetical protein |
| NODE_104_length_519_cov_237.194611 | FIG | CDS | 194 | 406 | . | - | 2 | ID=fig\|159.23.peg.23;Name=hypothetical protein |
| NODE_108_length_1081_cov_106.000923 | FIG | CDS | 128 | 1060 | . | - | 2 | ID=fig\|159.23.peg.24;Name=methyltransferase2C FkbM family domain protein |
| NODE_126_length_4701_cov_195.413315 | FIG | CDS | 361 | 717 | . | - | 1 | ID=fig\|159.23.peg.336;Name=DNA-damage-inducible protein d |
| NODE_126_length_4701_cov_195.413315 | FIG | CDS | 1049 | 1579 | . | + | 2 | ID=fig\|159.23.peg.337;Name=hypothetical protein |
| NODE_126_length_4701_cov_195.413315 | FIG | CDS | 1686 | 4187 | . | + | 0 | ID=fig\|159.23.peg.338;Name=Phage tail length tape-measure protein |
| NODE_126_length_4701_cov_195.413315 | FIG | CDS | 4257 | 4667 | . | - | 0 | ID=fig\|159.23.peg.339;Name=Micrococcal nuclease (thermonuclease) homologs |
| NODE_156_length_2948_cov_270.566833 | FIG | CDS | 182 | 904 | . | + | 2 | ID=fig\|159.23.peg.530;Name=hypothetical protein |
| NODE_156_length_2948_cov_270.566833 | FIG | CDS | 907 | 1368 | . | - | 1 | ID=fig\|159.23.peg.531;Name=hypothetical protein |
| NODE_156_length_2948_cov_270.566833 | FIG | CDS | 1365 | 2318 | . | - | 0 | ID=fig\|159.23.peg.532;Name=hypothetical protein |
| NODE_156_length_2948_cov_270.566833 | FIG | CDS | 2485 | 2862 | . | - | 1 | ID=fig\|159.23.peg.533;Name=hypothetical protein |
| NODE_16_length_14303_cov_481.098785 | FIG | CDS | 362 | 712 | . | - | 2 | ID=fig\|159.23.peg.632;Name=hypothetical protein |
| NODE_16_length_14303_cov_481.098785 | FIG | CDS | 709 | 1254 | . | - | 1 | ID=fig\|159.23.peg.633;Name=hypothetical protein |
| NODE_16_length_14303_cov_481.098785 | FIG | CDS | 1479 | 1604 | . | - | 0 | ID=fig\|159.23.peg.634;Name=hypothetical protein |
| NODE_16_length_14303_cov_481.098785 | FIG | CDS | 2030 | 3031 | . | + | 2 | ID=fig\|159.23.peg.635;Name=hypothetical protein |
| NODE_16_length_14303_cov_481.098785 | FIG | CDS | 2997 | 4016 | . | - | 0 | ID=fig\|159.23.peg.636;Name=hypothetical protein |
| NODE_16_length_14303_cov_481.098785 | FIG | CDS | 4345 | 4575 | . | - | 1 | ID=fig\|159.23.peg.637;Name=hypothetical protein |
| NODE_16_length_14303_cov_481.098785 | FIG | CDS | 4735 | 5055 | . | - | 1 | ID=fig\|159.23.peg.638;Name=hypothetical protein |
| NODE_16_length_14303_cov_481.098785 | FIG | CDS | 5052 | 5639 | . | - | 0 | ID=fig\|159.23.peg.639;Name=hypothetical protein |
| NODE_16_length_14303_cov_481.098785 | FIG | CDS | 5852 | 6415 | . | - | 2 | ID=fig\|159.23.peg.640;Name=hypothetical protein |
| NODE_16_length_14303_cov_481.098785 | FIG | CDS | 6428 | 6883 | . | - | 2 | ID=fig\|159.23.peg.641;Name=hypothetical protein |
| NODE_16_length_14303_cov_481.098785 | FIG | CDS | 6973 | 8388 | . | - | 1 | ID=fig\|159.23.peg.642;Name=hypothetical protein |
| NODE_16_length_14303_cov_481.098785 | FIG | CDS | 8485 | 9420 | . | - | 1 | ID=fig\|159.23.peg.643;Name=hypothetical protein |
| NODE_16_length_14303_cov_481.098785 | FIG | CDS | 9435 | 9605 | . | - | 0 | ID=fig\|159.23.peg.644;Name=hypothetical protein |
| NODE_16_length_14303_cov_481.098785 | FIG | CDS | 9598 | 10473 | . | - | 1 | ID=fig\|159.23.peg.645;Name=hypothetical protein |
| NODE_16_length_14303_cov_481.098785 | FIG | CDS | 10673 | 12112 | . | - | 2 | ID=fig\|159.23.peg.646;Name=FIG00437581: hypothetical protein |
| NODE_16_length_14303_cov_481.098785 | FIG | CDS | 12217 | 14325 | . | - | 1 | ID=fig\|159.23.peg.647;Name=Gene Transfer Agent terminase protein |
| NODE_175_length_1118_cov_115.193199 | FIG | CDS | 123 | 1022 | . | + | 0 | ID=fig\|159.23.peg.675;Name=hypothetical protein |
| NODE_188_length_1656_cov_257.953491 | FIG | CDS | 64 | 447 | . | - | 1 | ID=fig\|159.23.peg.695;Name=hypothetical protein |
| NODE_188_length_1656_cov_257.953491 | FIG | CDS | 440 | 763 | . | - | 2 | ID=fig\|159.23.peg.696;Name=hypothetical protein |
| NODE_188_length_1656_cov_257.953491 | FIG | CDS | 750 | 1178 | . | - | 0 | ID=fig\|159.23.peg.697;Name=hypothetical protein |
| NODE_188_length_1656_cov_257.953491 | FIG | CDS | 1181 | 1651 | . | - | 2 | ID=fig\|159.23.peg.698;Name=hypothetical protein |
| NODE_18_length_4543_cov_481.309265 | FIG | CDS | 344 | 1654 | . | + | 2 | ID=fig\|159.23.peg.699;Name=massive surface protein MspG |
| NODE_18_length_4543_cov_481.309265 | FIG | CDS | 1672 | 2283 | . | + | 1 | ID=fig\|159.23.peg.700;Name=hypothetical protein |
| NODE_18_length_4543_cov_481.309265 | FIG | CDS | 2300 | 3313 | . | + | 2 | ID=fig\|159.23.peg.701;Name=hypothetical protein |
| NODE_18_length_4543_cov_481.309265 | FIG | CDS | 3329 | 3706 | . | + | 2 | ID=fig\|159.23.peg.702;Name=hypothetical protein |
| NODE_18_length_4543_cov_481.309265 | FIG | CDS | 3707 | 4219 | . | + | 2 | ID=fig\|159.23.peg.703;Name=hypothetical protein |
| NODE_236_length_514_cov_256.167328 | FIG | CDS | 269 | 523 | . | + | 2 | ID=fig\|159.23.peg.991;Name=hypothetical protein |
| NODE_350_length_303_cov_550.346558 | FIG | CDS | 52 | 354 | . | - | 1 | ID=fig\|159.23.peg.1773;Name=hypothetical protein |
| NODE_428_length_858_cov_117.562935 | FIG | CDS | 200 | 739 | . | + | 2 | ID=fig\|159.23.peg.2001;Name=Phage protein |
| NODE_42_length_1569_cov_487.210327 | FIG | CDS | 208 | 492 | . | - | 1 | ID=fig\|159.23.peg.2002;Name=hypothetical protein |
| NODE_42_length_1569_cov_487.210327 | FIG | CDS | 600 | 806 | . | - | 0 | ID=fig\|159.23.peg.2003;Name=hypothetical protein |
| NODE_42_length_1569_cov_487.210327 | FIG | CDS | 791 | 973 | . | - | 2 | ID=fig\|159.23.peg.2004;Name=hypothetical protein |
| NODE_42_length_1569_cov_487.210327 | FIG | CDS | 966 | 1247 | . | - | 0 | ID=fig\|159.23.peg.2005;Name=hypothetical protein |
| NODE_46_length_2849_cov_232.349945 | FIG | CDS | 119 | 1027 | . | + | 2 | ID=fig\|159.23.peg.2048;Name=hypothetical protein |
| NODE_46_length_2849_cov_232.349945 | FIG | CDS | 1032 | 1472 | . | - | 0 | ID=fig\|159.23.peg.2049;Name=hypothetical protein |
| NODE_46_length_2849_cov_232.349945 | FIG | CDS | 1484 | 2062 | . | - | 2 | ID=fig\|159.23.peg.2050;Name=hypothetical protein |
| NODE_46_length_2849_cov_232.349945 | FIG | CDS | 2139 | 2642 | . | - | 0 | ID=fig\|159.23.peg.2051;Name=hypothetical protein |
| NODE_48_length_1665_cov_252.682877 | FIG | CDS | 39 | 296 | . | + | 0 | ID=fig\|159.23.peg.2052;Name=putative methyl-accepting chemotaxis sensory transducer |
| NODE_54_length_4182_cov_459.043518 | FIG | CDS | 743 | 1354 | . | - | 2 | ID=fig\|159.23.peg.2062;Name=hypothetical protein |
| NODE_54_length_4182_cov_459.043518 | FIG | CDS | 1351 | 2568 | . | - | 1 | ID=fig\|159.23.peg.2063;Name=hypothetical protein |
| NODE_54_length_4182_cov_459.043518 | FIG | CDS | 2574 | 2915 | . | - | 0 | ID=fig\|159.23.peg.2064;Name=hypothetical protein |
| NODE_54_length_4182_cov_459.043518 | FIG | CDS | 2918 | 3286 | . | - | 2 | ID=fig\|159.23.peg.2065;Name=hypothetical protein |
| NODE_54_length_4182_cov_459.043518 | FIG | CDS | 3287 | 4048 | . | - | 2 | ID=fig\|159.23.peg.2066;Name=hypothetical protein |
| NODE_60_length_3833_cov_531.471436 | FIG | CDS | 352 | 1002 | . | - | 1 | ID=fig\|159.23.peg.2091;Name=hypothetical protein |
| NODE_60_length_3833_cov_531.471436 | FIG | CDS | 1119 | 2696 | . | - | 0 | ID=fig\|159.23.peg.2092;Name=hypothetical protein |
| NODE_60_length_3833_cov_531.471436 | FIG | CDS | 2719 | 2883 | . | - | 1 | ID=fig\|159.23.peg.2093;Name=hypothetical protein |
| NODE_60_length_3833_cov_531.471436 | FIG | CDS | 2909 | 3139 | . | - | 2 | ID=fig\|159.23.peg.2094;Name=hypothetical protein |
| NODE_60_length_3833_cov_531.471436 | FIG | CDS | 3139 | 3348 | . | - | 1 | ID=fig\|159.23.peg.2095;Name=hypothetical protein |
| NODE_60_length_3833_cov_531.471436 | FIG | CDS | 3361 | 3516 | . | - | 1 | ID=fig\|159.23.peg.2096;Name=hypothetical protein |
| NODE_79_length_1535_cov_217.173294 | FIG | CDS | 111 | 242 | . | + | 0 | ID=fig\|159.23.peg.2430;Name=hypothetical protein |
| NODE_79_length_1535_cov_217.173294 | FIG | CDS | 272 | 814 | . | - | 2 | ID=fig\|159.23.peg.2431;Name=hypothetical protein |
| NODE_79_length_1535_cov_217.173294 | FIG | CDS | 839 | 1375 | . | - | 2 | ID=fig\|159.23.peg.2432;Name=hypothetical protein |
| NODE_80_length_4109_cov_548.463867 | FIG | CDS | 75 | 290 | . | + | 0 | ID=fig\|159.23.peg.2433;Name=hypothetical protein |
| NODE_80_length_4109_cov_548.463867 | FIG | CDS | 373 | 789 | . | + | 1 | ID=fig\|159.23.peg.2434;Name=hypothetical protein |
| NODE_80_length_4109_cov_548.463867 | FIG | CDS | 789 | 1142 | . | + | 0 | ID=fig\|159.23.peg.2435;Name=hypothetical protein |
| NODE_80_length_4109_cov_548.463867 | FIG | CDS | 1145 | 1306 | . | + | 2 | ID=fig\|159.23.peg.2436;Name=hypothetical protein |
| NODE_80_length_4109_cov_548.463867 | FIG | CDS | 1303 | 1524 | . | + | 1 | ID=fig\|159.23.peg.2437;Name=hypothetical protein |
| NODE_80_length_4109_cov_548.463867 | FIG | CDS | 1521 | 2030 | . | + | 0 | ID=fig\|159.23.peg.2438;Name=hypothetical protein |
| NODE_80_length_4109_cov_548.463867 | FIG | CDS | 2027 | 2215 | . | + | 2 | ID=fig\|159.23.peg.2439;Name=hypothetical protein |
| NODE_80_length_4109_cov_548.463867 | FIG | CDS | 2212 | 2883 | . | + | 1 | ID=fig\|159.23.peg.2440;Name=RNA polymerase sigma factor SigB |
| NODE_80_length_4109_cov_548.463867 | FIG | CDS | 2893 | 3081 | . | + | 1 | ID=fig\|159.23.peg.2441;Name=hypothetical protein |
| NODE_80_length_4109_cov_548.463867 | FIG | CDS | 3092 | 3274 | . | + | 2 | ID=fig\|159.23.peg.2442;Name=hypothetical protein |
| NODE_80_length_4109_cov_548.463867 | FIG | CDS | 3571 | 4035 | . | + | 1 | ID=fig\|159.23.peg.2443;Name=hypothetical protein |

Table C. Number of full length genes (90% coverage of to *B.hyodysenteriae* WA1) in each strain found at specific alignment sequence percent identities.

|  | 50% | 60% | 70% | 80% | 90% | 95% | 100% |
| --- | --- | --- | --- | --- | --- | --- | --- |
| Q17 | 2,516 | 2,509 | 2,498 | 2,493 | 2,468 | 2,409 | 1,869 |
| Vic2 | 2,509 | 2,500 | 2,493 | 2,481 | 2,442 | 2,364 | 1,690 |
| NSW5 | 2,466 | 2,454 | 2,446 | 2,433 | 2,367 | 2,257 | 1,332 |
| ST210 | 2,420 | 2,409 | 2,396 | 2,368 | 2,276 | 2,100 | 750 |
| ST190 | 2,417 | 2,407 | 2,397 | 2,379 | 2,280 | 2,092 | 743 |
| 865 | 2,373 | 2,360 | 2,348 | 2,331 | 2,210 | 1,982 | 583 |
| G21 | 2,365 | 2,350 | 2,341 | 2,317 | 2,194 | 1,950 | 576 |
| B8044 | 2,371 | 2,360 | 2,348 | 2,320 | 2,210 | 1,982 | 562 |
| NX | 2,310 | 2,300 | 2,286 | 2,267 | 2,146 | 1,928 | 546 |
| G44 | 2,352 | 2,341 | 2,320 | 2,288 | 2,179 | 1,965 | 540 |
| NSW15 | 2,328 | 2,308 | 2,296 | 2,269 | 2,161 | 1,926 | 539 |
| ST195 | 2,357 | 2,343 | 2,334 | 2,306 | 2,200 | 1,989 | 533 |
| FMV89.3323 | 2,356 | 2,343 | 2,335 | 2,310 | 2,192 | 1,974 | 532 |
| WA100 | 2,337 | 2,322 | 2,310 | 2,283 | 2,165 | 1,945 | 528 |
| FM88.90 | 2,327 | 2,309 | 2,299 | 2,275 | 2,149 | 1,929 | 517 |
| B78^T^ | 2,363 | 2,350 | 2,341 | 2,317 | 2,192 | 1,957 | 509 |
| B204 | 2,367 | 2,355 | 2,338 | 2,306 | 2,189 | 1,967 | 504 |
| B6933 | 2,393 | 2,381 | 2,367 | 2,345 | 2,232 | 2,001 | 487 |
| ST265 | 2,332 | 2,321 | 2,304 | 2,276 | 2,160 | 1,938 | 479 |

Table D. Number of predicted genes in each strain that matched with the KEGG database.

|  | KEGG CDS MATCHES |
| --- | --- |
| 865 | 636 |
| FMV89.3323 | 636 |
| NX | 637 |
| B204 | 640 |
| B6933 | 649 |
| B78^T^ | 641 |
| B8044 | 612 |
| G21 | 660 |
| G44 | 631 |
| NSW15 | 632 |
| NSW5 | 645 |
| Q17 | 639 |
| ST190 | 640 |
| ST195 | 638 |
| ST210 | 642 |
| ST265 | 639 |
| Vic2 | 644 |
| WA100 | 637 |
